# Theoretical analysis of inducer and operator binding for cyclic-AMP receptor protein mutants

**DOI:** 10.1371/journal.pone.0204275

**Published:** 2018-09-26

**Authors:** Tal Einav, Julia Duque, Rob Phillips

**Affiliations:** 1 Department of Physics, California Institute of Technology, Pasadena, CA, United States of America; 2 Department of Physics and the London Centre for Nanotechnology, University College London, London, United Kingdom; 3 Division of Biology and Biological Engineering, California Institute of Technology, Pasadena, CA, United States of America; 4 Department of Applied Physics, California Institute of Technology, Pasadena, CA, United States of America; Universitat Pompeu Fabra, SPAIN

## Abstract

Allosteric transcription factors undergo binding events at inducer binding sites as well as at distinct DNA binding domains, and it is difficult to disentangle the structural and functional consequences of these two classes of interactions. We compare the ability of two statistical mechanical models—the Monod-Wyman-Changeux (MWC) and the Koshland-Némethy-Filmer (KNF) models of protein conformational change—to characterize the multi-step activation mechanism of the broadly acting cyclic-AMP receptor protein (CRP). We first consider the allosteric transition resulting from cyclic-AMP binding to CRP, then analyze how CRP binds to its operator, and finally investigate the ability of CRP to activate gene expression. We use these models to examine a beautiful recent experiment that created a single-chain version of the CRP homodimer, creating six mutants using all possible combinations of the wild type, D53H, and S62F subunits. We demonstrate that the MWC model can explain the behavior of all six mutants using a small, self-consistent set of parameters whose complexity scales with the number of subunits, providing a significant benefit over previous models. In comparison, the KNF model not only leads to a poorer characterization of the available data but also fails to generate parameter values in line with the available structural knowledge of CRP. In addition, we discuss how the conceptual framework developed here for CRP enables us to not merely analyze data retrospectively, but has the predictive power to determine how combinations of mutations will interact, how double mutants will behave, and how each construct would regulate gene expression.

## Introduction

Transcriptional regulation lies at the heart of cellular decision making, and understanding how cells modify the myriad of players involved in this process remains challenging. The cyclic-AMP receptor protein (CRP; also known as the catabolite receptor protein, CAP) is an allosteric transcription factor that regulates over 150 genes in *Escherichia coli* [1–4]. Upon binding to cyclic-AMP (cAMP), the homodimeric CRP undergoes a conformational change whereby two alpha helices reorient to open a DNA binding domain [5], allowing CRP to bind to DNA and affect transcription [6–8]. While much is known about the molecular details of CRP and how different mutations modify its functionality [9, 10], each new CRP mutant is routinely analyzed in isolation using phenomenological models. We argue that given the hard-won structural insights into the conformational changes of proteins like CRP, it is important to test how well mechanistically motivated models of such proteins can characterize the wealth of available data.

**Fig 1:**
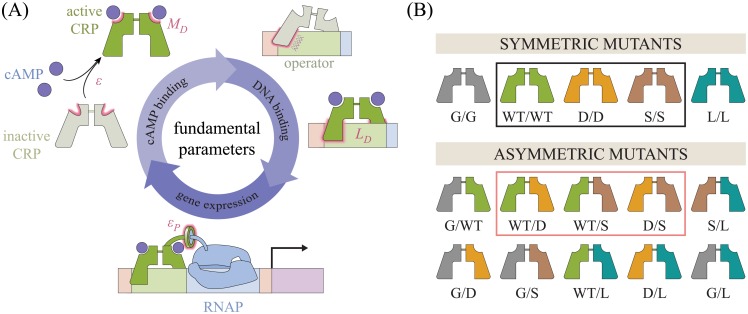
Key parameters governing CRP function. (A) Within the MWC and KNF models, each CRP subunit can assume either an active or an inactive conformation with a free energy difference *ϵ* between the two states. cAMP can bind to CRP (with a dissociation constant $M_{D}^{A}$ in the active state and $M_{D}^{I}$ in the inactive state) and promotes the active state ($M_{D}^{A}<M_{D}^{I}$ in the MWC model; $M_{D}^{I}\to \infty$ in the KNF model). Active CRP has a higher affinity for the operator ($L_{D}^{A}$) than the inactive state ($L_{D}^{I}$). When CRP is bound to DNA, it promotes RNA polymerase binding through an interaction energy *ϵ*_*P*_, thereby enhancing gene expression. (B) Lanfranco *et al*. constructed a single-chain CRP molecule whose two subunits could be mutated independently. All possible dimers are shown using five mutant subunits: wild type (WT), D (D53H), S (S62F), G (G141Q), and L (L148R). Lanfranco *et al*. constructed the six mutants comprised of WT, D, and S (black and pink boxes) and analyzed each mutant independently.

The picture that has emerged from various domains of biology is that allostery involves the interplay of a spectrum of dynamically linked states [11–17]. In some systems, it is straightforward to partition these states into the physiologically relevant categories; for example, CRP naturally divides into the cAMP unbound, singly bound, and doubly bound states as well as the DNA bound and unbound states. Nuclear magnetic resonance (NMR) and isothermal titration calorimetry (ITC) have begun to tease out the precise thermodynamics of the underlying interactions between these states [18, 19]. These methods have demonstrated that allosteric regulation in CRP includes both large structural changes as well as entropic modifications that make the protein more rigid [20, 21]. In this work, we ask whether we can capitalize upon this detailed knowledge of the system to construct a coarse-grained model of the multi-step activation cycle of CRP shown in [Fig 1(A) using a compact set of parameters. Specifically, we investigate variants of the Monod-Wyman-Changeux (MWC) model, which posits that both CRP subunits fluctuate concurrently between an active and inactive conformational state [22], and the Koshland-Némethy-Filmer (KNF) model, which proposes that each subunit must independently transition from an inactive to active state upon ligand binding [23], adapted for the CRP system. These two models have been investigated in a wide variety of allosteric systems, and evidence for both models as well as their shortcomings have been extensively analyzed [24–28]. Nevertheless, the simple thermodynamic view provided by the MWC and KNF models provides fertile ground to both verify how well we understand the critical factors governing CRP behavior as well as to explore hypotheses about mutational perturbations to the system.

Our paper is inspired by a recent *in vitro* study of CRP performed by Lanfranco *et al*. who engineered a single-chain CRP molecule whose two subunits are tethered together by an unstructured polypeptide linker [29]. This construct enabled them to mutate each subunit independently, providing a novel setting within which to analyze the combinatorial effects of mutations. Specifically, they took three distinct CRP subunits—the wild type (WT) subunit and the well characterized mutations D53H and S62F (denoted D and S, respectively) originally chosen to perturb the transcription factor’s cAMP binding domain [30, 31]—and linked them together in every possible combination to create six CRP mutants as shown in Fig 1(B) (black and pink boxes). Lanfranco *et al*. measured the cAMP-binding and DNA-binding capabilities of these mutants, separating these two key components of transcription factor activation. In this work, we present an analysis of these CRP mutants that demonstrates how their diverse phenotypes are related by their subunit compositions.

More specifically, the effects of mutations are often difficult to interpret, and indeed the results from Lanfranco *et al*. showed no clear pattern. The behavior of each mutant was analyzed *independently* by fitting its binding curve to a second order polynomial [29]. In this work, we propose an alternative framework that bolsters our understanding of the system in two significant ways: (1) we link the response functions of each CRP construct to its subunit composition, closing the gap between structure and function and (2) the number of parameters in our model scales linearly with the number of subunits whereas the number of parameters in the original analysis scaled with the number of CRP mutants (i.e. the square of the number of subunits). The advantage of this scaling behavior grows with the number of subunits. For example, this work focuses on the CRP mutants made by Lanfranco *et al*. using three subunits (black and pink boxes in Fig 1(B)). If we include two additional well-characterized mutants—such as G141Q (G) and L148R (L) [32]—for a total of *N* = 5 subunits, our model would only require 2*N* = 10 parameters to describe the $\frac{N(N+1)}{2}=15$ mutants whereas a model analyzing each mutant independently would require 30 parameters (2 per mutant). With *N* = 10 subunits, we would require 20 parameters to understand 55 mutants while a model characterizing individual mutants would require 110 parameters.

In addition to analyzing the available *in vitro* data for these mutants, we consider how each construct would promote gene expression *in vivo*. Because CRP is a global activator, its activity within the cell is tightly regulated by enzymes that produce, degrade, and actively transport cAMP [7]. We discuss how these processes can either be modeled theoretically or excised experimentally and calibrate our resulting framework for transcription using gene expression measurements for wild type CRP. In this manner, we find a small, self-consistent set of parameters able to characterize each step of CRP activation shown in Fig 1(A).

The remainder of this paper is organized as follows. First, we characterize the interaction between cAMP and CRP for the six CRP mutants created by Lanfranco *et al*. and quantify the key parameters governing this behavior. Next, we analyze the interaction between CRP and DNA and discuss how the inferred parameters align with structural knowledge of the system. Finally, we consider how CRP enhances gene expression and extend the results from Lanfranco *et al*. to predict the activation profiles of the CRP mutants within a cellular environment.

## Results

### The interaction between CRP and cAMP

In this section, we examine the cAMP-CRP binding process through the lenses of generalized MWC and KNF models which tie each mutant’s behavior to its subunit composition. We find that both frameworks can characterize data from a suite of CRP mutants using a compact set of parameters, but only the interpretation of the MWC parameters is consistent with structural knowledge of CRP.

#### MWC model

**Fig 2:**
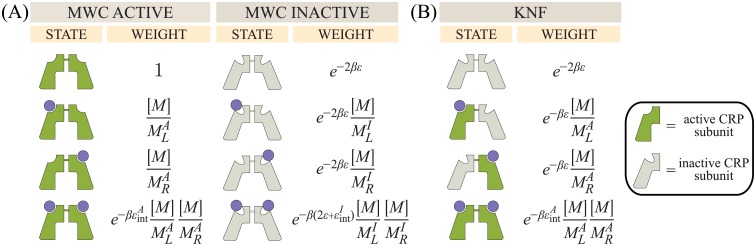
Macroscopic states and Boltzmann weights for cAMP binding to CRP. (A) Within the MWC model, cAMP (purple circles) may bind to a CRP subunit in either the active (dark green) or inactive (light green) state. $M_{L}^{A}$ and $M_{L}^{I}$ represent the dissociation constants of the left subunit in the active and inactive states, respectively, while $M_{R}^{A}$ and $M_{R}^{I}$ represent the analogous dissociation constants for the right subunit. [*M*] denotes the concentration of cAMP and *ϵ* represents the free energy difference between each subunit’s inactive and active states with $\beta =\frac{1}{k_{B}T}$. $\epsilon_{\text{int}}^{A}$ and $\epsilon_{\text{int}}^{I}$ represent a cooperative energy when two cAMP are bound to CRP in the active and inactive states, respectively. (B) The KNF model assumes that the two CRP subunits are inactive when unbound to cAMP and transition to the active state immediately upon binding to cAMP. The parameters have the same meaning as in the MWC model, but states where one subunit is active while the other is inactive are allowed.

We first formulate a description of cAMP-CRP binding using a generalized form of the MWC model, where the two subunits of each CRP molecule fluctuate concurrently between an active and inactive state. The different conformations of CRP binding to cAMP and their corresponding Boltzmann weights are shown in Fig 2(A). We define the free energy difference between inactive CRP and active CRP as 2*ϵ* (or *ϵ* per subunit). *ϵ* will be large and negative since the activator is preferentially inactive in the absence of ligand, which will allow us to simplify the description of the system (see S1 Text Section A). $\beta =\frac{1}{k_{B}T}$ where *k*_*B*_ is Boltzmann’s constant and *T* represents temperature. The two cAMP binding events are known to be cooperative [26, 33, 34], where the magnitude and the sign of this cooperativity (whether it is favorable or unfavorable) strongly depends upon the conditions of the buffer, mutational perturbations to the system, and whether the full or partial CRP protein is considered [29, 35, 36]. To that end, we introduce two types of cooperativity. First, the classic MWC model is inherently cooperative, as the binding of each ligand alters the probable conformation and hence binding affinity of the other binding site; however, this mode of cooperativity can only be favorable [37]. Because CRP may also exhibit negative cooperativity, we introduce explicit interaction energies $\epsilon_{\text{int}}^{A}$ and $\epsilon_{\text{int}}^{I}$ between two ligands in the active and inactive CRP states, respectively. For simplicity, and because it will enable us to characterize the CRP collectively rather than requiring a unique parameter for each mutant, we assume that these explicit cooperative interactions are the same across all constructs (see S1 Text Section B where we relax such assumptions).

For each cAMP-CRP dissociation constant $M_{X}^{Y}$, the subscript denotes which CRP subunit it describes—either the left (*L*) or right (*R*) subunit—while the superscript denotes the active (*A*) or inactive (*I*) state of CRP. Note that the left and right subunits may be different (see Fig 1(B)). Given a cAMP concentration [*M*], the fraction of occupied cAMP binding sites is given by

$$\text{fractional}\;\text{CRP}\;\text{occupancy}([M])=\frac{\frac{1}{2}(\frac{\left[M\right]}{M_{L}^{A}}+\frac{\left[M\right]}{M_{R}^{A}})+e^{-\beta \epsilon_{\text{int}}^{A}}\frac{\left[M\right]}{M_{L}^{A}}\frac{\left[M\right]}{M_{R}^{A}}+e^{-2\beta \epsilon }(\frac{1}{2}(\frac{\left[M\right]}{M_{L}^{I}}+\frac{\left[M\right]}{M_{R}^{I}})+e^{-\beta \epsilon_{\text{int}}^{I}}\frac{\left[M\right]}{M_{L}^{I}}\frac{\left[M\right]}{M_{R}^{I}})}{1+\frac{\left[M\right]}{M_{L}^{A}}+\frac{\left[M\right]}{M_{R}^{A}}+e^{-\beta \epsilon_{\text{int}}^{A}}\frac{\left[M\right]}{M_{L}^{A}}\frac{\left[M\right]}{M_{R}^{A}}+e^{-2\beta \epsilon }(1+\frac{\left[M\right]}{M_{L}^{I}}+\frac{\left[M\right]}{M_{R}^{I}}+e^{-\beta \epsilon_{\text{int}}^{I}}\frac{\left[M\right]}{M_{L}^{I}}\frac{\left[M\right]}{M_{R}^{I}})}.$$ (1)

Here, the fractional occupancy of CRP bound to zero, one, or two cAMP equals 0, ½, and 1, respectively. Experimentally, the fractional occupancy was measured *in vitro* in the absence of DNA using ANS fluorescence which utilizes a fluorescent probe triggered by the conformational change of cAMP binding to CRP [29].

**Fig 3:**
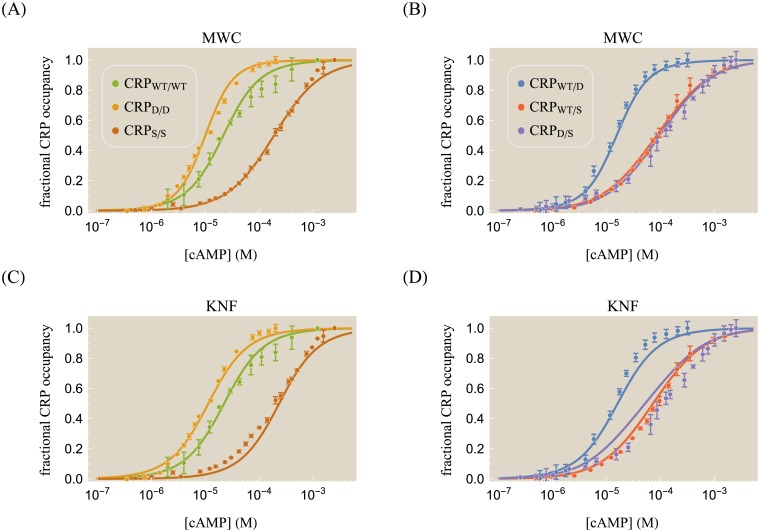
cAMP binding for different CRP mutants. In addition to the wild type CRP subunit (denoted WT), the mutation D53H (denoted D) and the mutation S62F (denoted S) can be applied to either subunit as indicated by the subscripts in the legend. (A) Curves were characterized using the MWC model, Eq (1). The D subunit increases CRP’s affinity for cAMP while the S subunit decreases this affinity. (B) Asymmetrically mutating the two subunits results in distinct cAMP binding curves. The data for the WT/D mutant lies between the WT/WT and D/D data in Panel A, and analogous statements apply for the WT/S and D/S mutants. (C) The fraction of CRP in the active state. Within the MWC model, mutants with an S subunit will be inactive even in the limit of saturating cAMP. (D) The symmetric and (E) asymmetric mutants can also be analyzed using the KNF model, Eq (6), resulting in curves that are similar to those found by the MWC model. (F) The KNF model predicts that all CRP mutants will be completely active in the limit of saturating cAMP. The (corrected) sample standard deviation $\sqrt{\frac{1}{n-1}\sum_{j=1}^{n}{\left(y_{\text{theory}}^{\left(j\right)}-y_{\text{data}}^{\left(j\right)}\right)}^{2}}$ equals 0.03 for the MWC model and 0.05 for the KNF model, and the best-fit parameters for both models are given in Table 1. Data reproduced from Ref. [29].

Lanfranco *et al*. considered CRP subunits with either the D53H or S62F point mutations (hereafter denoted by D and S, respectively), with the D subunit binding more strongly to cAMP than the wild type while the S subunit binds more weakly as shown in Fig 3(A). While we could characterize the dose-response curves of each CRP mutant independently—for example, by using Eq (1) to extract a set of parameters for each mutant—such an analysis lacks a direct connection between the subunit composition and the corresponding binding behavior. Instead, we assume that the cAMP binding affinity for each subunit should be uniquely dictated by that subunit’s identity as either the WT, D, or S subunit. To that end, we represent the fractional occupancy of CRP_D/WT_ using Eq (1) with one D subunit ($M_{L}^{A}=M_{\text{D}}^{A}$, $M_{L}^{I}=M_{\text{D}}^{I}$) and one WT subunit ($M_{R}^{A}=M_{\text{WT}}^{A}$, $M_{R}^{I}=M_{\text{WT}}^{I}$). The equations for the remaining CRP mutants follow analogously, tying the behavior of each mutant to its subunit composition. For simplicity, we will assume that the D and S mutations do not alter the cAMP interaction energies $\epsilon_{\text{int}}^{A}$ and $\epsilon_{\text{int}}^{I}$.

**Table 1:** Parameters for cAMP binding to CRP. The data in Fig 3 can be characterized using a single set of dissociation constants for the WT, D, and S subunits whose values and standard errors are shown. To excise parameter degeneracy, the active-inactive free energy difference *ϵ* and the cAMP interaction energy in the active state $\epsilon_{\text{int}}^{A}$ are absorbed into the active state dissociation constants in the MWC model (Eqs (2) and (3)). Similarly, *ϵ* is absorbed into the KNF dissociation constants (Eqs (6) and (7)).

MWC Parameter	Best-Fit Value	KNF Parameter	Best-Fit Value
${\tilde{M}}_{\text{WT}}^{A}$, $M_{\text{WT}}^{I}$	{25 ± 1, 40 ± 3} × 10^−6^ M	${\bar{M}}_{\text{WT}}^{A}$	(30 ± 2) × 10^−6^ M
${\tilde{M}}_{\text{D}}^{A}$, $M_{\text{D}}^{I}$	{10 ± 1, 50 ± 5} × 10^−6^ M	${\bar{M}}_{\text{D}}^{A}$	(20 ± 1) × 10^−6^ M
${\tilde{M}}_{\text{S}}^{A}$, $M_{\text{S}}^{I}$	{≥ 1000, 200 ± 10} × 10^−6^ M	${\bar{M}}_{\text{S}}^{A}$	(350 ± 10) × 10^−6^ M
K˜ϵintI	0.0 ± 0.2*k*_*B*_ *T*	$\epsilon_{\text{int}}^{A}$	−0.8 ± 0.2*k*_*B*_ *T*

One difficulty in inferring parameter values from Eq (1) is that degenerate sets of parameters may produce equivalent binding curves. For example, in S1 Text Section A, we demonstrate how the same cAMP-CRP binding curves can arise from an arbitrarily large and negative free energy difference (*ϵ* → − ∞) provided that the dissociation constants scale appropriately. In that same supporting information section, we demonstrate how this degeneracy can be excised so that Eq (1) is well approximated by the following form,

$$\begin{array}{c} \text{fractional}\;\text{CRP}\;\text{occupancy}\left(\left[M\right]\right)\approx \frac{\frac{\left[M\right]}{{\tilde{M}}_{L}^{A}}\frac{\left[M\right]}{{\tilde{M}}_{R}^{A}}+\frac{1}{2}(\frac{\left[M\right]}{M_{L}^{I}}+\frac{\left[M\right]}{M_{R}^{I}})+e^{-\beta \epsilon_{\text{int}}^{I}}\frac{\left[M\right]}{M_{L}^{I}}\frac{\left[M\right]}{M_{R}^{I}}}{\frac{\left[M\right]}{{\tilde{M}}_{L}^{A}}\frac{\left[M\right]}{{\tilde{M}}_{R}^{A}}+(1+\frac{\left[M\right]}{M_{L}^{I}}+\frac{\left[M\right]}{M_{R}^{I}}+e^{-\beta \epsilon_{\text{int}}^{I}}\frac{\left[M\right]}{M_{L}^{I}}\frac{\left[M\right]}{M_{R}^{I}})}, \end{array}$$ (2)

where we have neglected the unbound and singly-cAMP-bound active CRP states and defined the effective dissociation constants

$$\begin{array}{c} {\tilde{M}}_{L}^{A}=e^{-\beta \epsilon }e^{\beta \epsilon_{\text{int}}^{A}/2}M_{L}^{A} \end{array}$$ (3)

and

$$\begin{array}{c} {\tilde{M}}_{R}^{A}=e^{-\beta \epsilon }e^{\beta \epsilon_{\text{int}}^{A}/2}M_{R}^{A}. \end{array}$$ (4)

Using Eq (2), we can extract the set of effective dissociation constants for the WT, D, and S subunits that determine the behavior of all six CRP mutants. The resulting parameters (shown in Table 1) give rise to the cAMP-CRP binding curves in Fig 3(A) and 3(B). Note that in removing the parameter degeneracy using Eqs (3) and (4), we can no longer determine the individual values of *ϵ*, $\epsilon_{\text{int}}^{A}$, and the active state dissociation constants $M_{X}^{A}$, but rather only the parameter combinations ${\tilde{M}}_{X}^{A}$. On the other hand, the inactive state cooperativity energy $\epsilon_{\text{int}}^{I}$ can be unambiguously determined to be negligible. The effective dissociation constant of the S subunit in the MWC model can only be bounded from below as ${\tilde{M}}_{\text{S}}^{A}\geq 1000\times 10^{-6}\;\text{M}$. However, NMR measurements reported that in the limit of saturating cAMP, the S/S mutant will be inactive state 98% of the time (see Fig 3(C) and S1 Text Section B) which corresponds to a value of ${\tilde{M}}_{\text{S}}^{A}\approx 1300\times 10^{-6}\;\text{M}$ [20].

In S1 Text Section B, we demonstrate that the symmetric CRP mutants in Fig 3(A) provide sufficient information to approximate the behavior of the asymmetric mutants in Fig 3(B). We further show that fitting each CRP data set individually to the MWC or KNF models without constraining the WT, D, and S subunits to a single unified set of dissociation constants results in only a marginal improvement over the constrained fitting. Finally, we analyze the slope of each cAMP binding response and explain why they are nearly identical for the six CRP mutants. In S1 Text Section C, we investigate the effects of the double mutation D+S on a single subunit by comparing its CRP occupancy data supposing that the change in free energy from both mutations is additive and independent. Within this epistasis-free model, we can similarly predict the behavior of other double mutants including CRP_D/D+S_, CRP_S/D+S_, and CRP_D+S/D+S_.

Lastly, we reiterate that the MWC model presented here provides a coarse-grained model of the system. For example, experiments have revealed that the first cAMP binding does not alter the conformation of the second subunit, although it does drastically diminish its protein motions [34]. In the MWC model, these effects are captured both by the inherent cooperativity [37] as well as by the explicit interaction energies $\epsilon_{\text{int}}^{A}$ and $\epsilon_{\text{int}}^{I}$, since within this model the binding of one cAMP can induce the other CRP subunit to change (e.g. changing the unbound inactive state into the active singly-bound state). In light of these results, we next consider an alternative model of the system which explicitly assumes that each subunit only becomes active upon ligand binding.

#### KNF model

We now turn to a KNF analysis of CRP, where the two subunits are individually inactive when not bound to cAMP and become active upon binding as shown in Fig 2(B). Some studies have claimed that cAMP binding to one CRP subunit does not affect the state of the other subunit, in support of the KNF model [34]. Other studies, meanwhile, have reported that a fraction of CRP molecules are active even in the absence of cAMP, thereby favoring an MWC interpretation [9, 38]. To determine whether either model can accurately represent the system, we explore some of the consequences of a KNF interpretation of CRP.

Using the statistical mechanical states of the system in Fig 2(B), the occupancy of CRP is given by

$$\begin{array}{c} \text{fractional}\;\text{CRP}\;\text{occupancy}\left(\left[M\right]\right)=\frac{\frac{e^{-\beta \epsilon }}{2}(\frac{\left[M\right]}{M_{L}^{A}}+\frac{\left[M\right]}{M_{R}^{A}})+e^{-\beta \epsilon_{\text{int}}^{A}}\frac{\left[M\right]}{M_{L}^{A}}\frac{\left[M\right]}{M_{R}^{A}}}{e^{-2\beta \epsilon }+e^{-\beta \epsilon }(\frac{\left[M\right]}{M_{L}^{A}}+\frac{\left[M\right]}{M_{R}^{A}})+e^{-\beta \epsilon_{\text{int}}^{A}}\frac{\left[M\right]}{M_{L}^{A}}\frac{\left[M\right]}{M_{R}^{A}}}. \end{array}$$ (5)

where the parameters have the same meaning as in the MWC model. Multiplying the numerator and denominator by *e*^2*βϵ*^, we obtain the form

$$\begin{array}{c} \text{fractional}\;\text{CRP}\;\text{occupancy}\left(\left[M\right]\right)=\frac{\frac{1}{2}(\frac{\left[M\right]}{{\bar{M}}_{L}^{A}}+\frac{\left[M\right]}{{\bar{M}}_{R}^{A}})+e^{-\beta \epsilon_{\text{int}}^{A}}\frac{\left[M\right]}{{\bar{M}}_{L}^{A}}\frac{\left[M\right]}{{\bar{M}}_{R}^{A}}}{1+\frac{\left[M\right]}{{\bar{M}}_{L}^{A}}+\frac{\left[M\right]}{{\bar{M}}_{R}^{A}}+e^{-\beta \epsilon_{\text{int}}^{A}}\frac{\left[M\right]}{{\bar{M}}_{L}^{A}}\frac{\left[M\right]}{{\bar{M}}_{R}^{A}}} \end{array}$$ (6)

where, similar to the MWC model effective dissociation constants Eqs (3) and (4), we have defined

$$\begin{array}{c} {\bar{M}}_{L}^{A}=e^{-\beta \epsilon }M_{L}^{A} \end{array}$$ (7)

and

$$\begin{array}{c} {\bar{M}}_{R}^{A}=e^{-\beta \epsilon }M_{R}^{A}. \end{array}$$ (8)

This simplification occurs because within the KNF model, a CRP monomer only switches from the inactive to active state upon cAMP binding. As a result, the free energy of cAMP binding to CRP and the free energy of the CRP undergoing its inactive-to-active state conformational always occur concurrently and may be combined into the effective dissociation constants ${\bar{M}}_{L}^{A}$ and ${\bar{M}}_{R}^{A}$.

As shown in Fig 3(D) and 3(E), the KNF model can approximately characterize the six mutant CRP binding curves, although the S/S and WT/D responses lie slightly below the data while the D/S curve deviates above the data. These discrepancies could potentially be alleviated by letting the interaction energy $\epsilon_{\text{int}}^{A}$ vary with each mutant, although doing so would significantly increase the number of parameters in the model (which would then scale with the number of mutants rather than the number of subunits). However, a greater failing of the KNF model is that it predicts that at saturating cAMP concentrations the protein will always be completely active, even though the S/S mutant is 98% inactive in this limit (Fig 3(F)) [20]. These results suggest that a more complex variant of the KNF model should be used to quantitatively dissect the CRP system.

### The interaction between CRP and DNA

We now turn to the second binding interaction experienced by CRP, namely, that between CRP and DNA. Since the preceding analysis demonstrated that the KNF model considered here cannot characterize the existing data, we proceed by only analyzing the MWC model.

**Fig 4:**
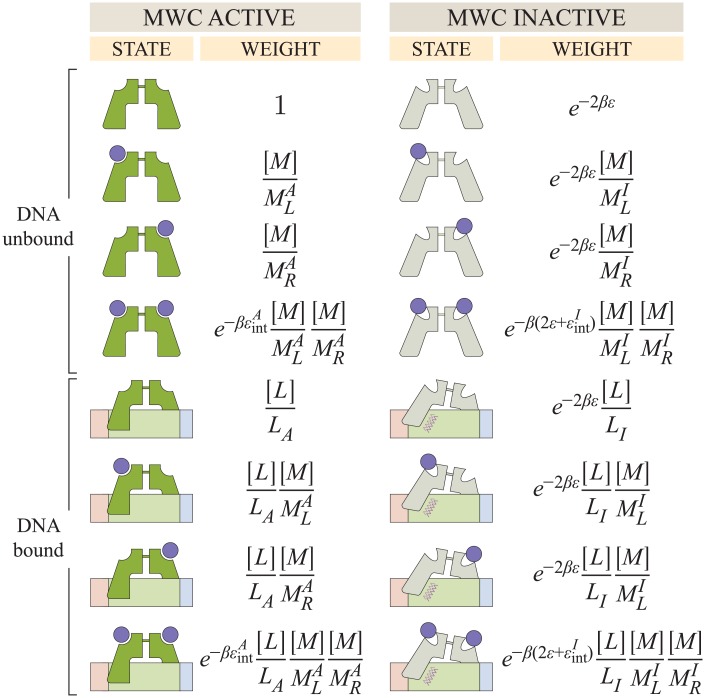
States and weights for CRP binding to DNA. The DNA unbound states from Fig 2 are shown together with the DNA bound states. The Boltzmann weight of each DNA bound state is proportional to the concentration [*L*] of CRP and inversely proportional to the CRP-DNA dissociation constants *L*_*A*_ or *L*_*I*_ for the active and inactive states, respectively.

Consider a concentration [*L*] of CRP whose subunits either assume an active state (where they tightly bind to DNA with a dissociation constant *L*_*A*_) or in an inactive state (characterized by weaker DNA binding with dissociation constant *L*_*I*_ satisfying *L*_*I*_ > *L*_*A*_). The states and weights of this system within the generalized MWC model are shown in Fig 4.

**Fig 5:**
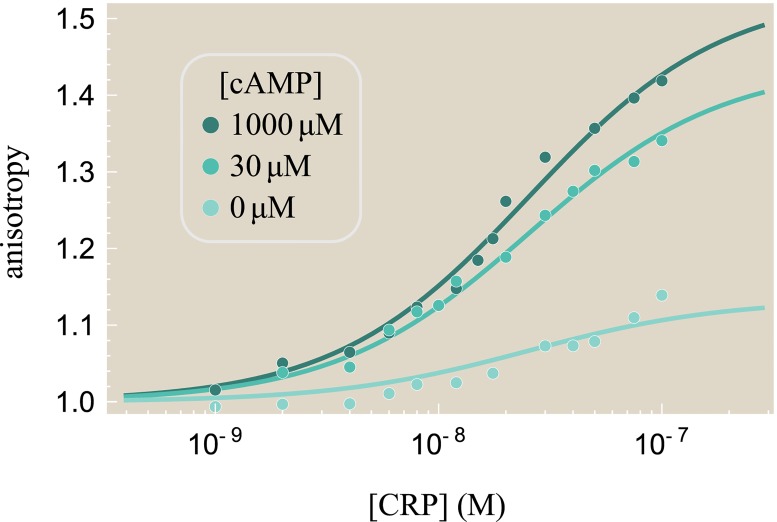
The interaction between CRP and DNA. Anisotropy of 32-bp fluorescein-labeled *lac* promoter binding to CRP_D/S_ at different concentrations of cAMP. An anisotropy of 1 corresponds to unbound DNA while higher values imply that DNA is bound to CRP. In the presence of cAMP, more CRP subunits will be active, and hence there will be greater anisotropy for any given concentration of CRP. The sample standard deviation $\sqrt{\frac{1}{n-1}\sum_{j=1}^{n}{\left(y_{\text{theory}}^{\left(j\right)}-y_{\text{data}}^{\left(j\right)}\right)}^{2}}$ is 0.01, with the corresponding parameters given in Tables 1 and 2. Data reproduced from Ref. [29].

Lanfranco *et al*. fluorescently labeled a short, 32 bp DNA sequence which binds to CRP. Using a spectrometer, they measured the anisotropy of this fluorescence when different concentrations of CRP and cAMP were added *in vitro* [29]. The data are shown in Fig 5(A) for CRP_D/S_ for various concentrations of the receptor and effector. When CRP binds, it slows the random tumbling of the DNA so that over very short time scales the fluorescence is oriented along a particular axis, resulting in a larger anisotropy readout. Unbound DNA is defined as having anisotropy equal to 1 while DNA-bound CRP with 0, 1, or 2 bound cAMP have higher anisotropies of 1 + *r*_0_, 1 + *r*_1_, and 1 + *r*_2_, respectively. Thus, the total anisotropy within the model is given by the weighted sum of each species [39], namely,

$$\begin{array}{c} \text{anisotropy}=1+r_{0}p_{0}+r_{1}p_{1}+r_{2}p_{2}. \end{array}$$ (9)

Here, *p*_0_, *p*_1_, and *p*_2_ represent the probabilities that DNA-bound CRP will be bound to 0, 1, and 2 cAMP molecules, respectively. Using the effective dissociation constants (Eqs (3) and (4)) and neglecting all terms proportional to the small quantity *e*^*βϵ*^, we can write these probabilities as

$$\begin{array}{c} p_{0}=\frac{e^{2\beta \epsilon }\frac{\left[L\right]}{L_{A}}+\frac{\left[L\right]}{L_{I}}}{Z}\approx \frac{\frac{\left[L\right]}{L_{I}}}{Z}, \end{array}$$ (10)

$$\begin{array}{c} p_{1}=\frac{e^{2\beta \epsilon }\frac{\left[L\right]}{L_{A}}(\frac{\left[M\right]}{M_{L}^{A}}+\frac{\left[M\right]}{M_{R}^{A}})+\frac{\left[L\right]}{L_{I}}(\frac{\left[M\right]}{M_{L}^{I}}+\frac{\left[M\right]}{M_{R}^{I}})}{Z}\approx \frac{\frac{\left[L\right]}{L_{I}}(\frac{\left[M\right]}{M_{L}^{I}}+\frac{\left[M\right]}{M_{R}^{I}})}{Z}, \end{array}$$ (11)

and

$$\begin{array}{c} p_{2}=\frac{e^{2\beta \epsilon }e^{-\beta \epsilon_{\text{int}}^{A}}\frac{\left[L\right]}{L_{A}}\frac{\left[M\right]}{M_{L}^{A}}\frac{\left[M\right]}{M_{R}^{A}}+e^{-\beta \epsilon_{\text{int}}^{I}}\frac{\left[L\right]}{L_{I}}\frac{\left[M\right]}{M_{L}^{I}}\frac{\left[M\right]}{M_{R}^{I}}}{Z}\approx \frac{\frac{\left[L\right]}{L_{A}}\frac{\left[M\right]}{{\tilde{M}}_{L}^{A}}\frac{\left[M\right]}{{\tilde{M}}_{R}^{A}}+e^{-\beta \epsilon_{\text{int}}^{I}}\frac{\left[L\right]}{L_{I}}\frac{\left[M\right]}{M_{L}^{I}}\frac{\left[M\right]}{M_{R}^{I}}}{Z} \end{array}$$ (12)

with

$$\begin{array}{cc} Z & =e^{2\beta \epsilon }(1+\frac{\left[L\right]}{L_{A}})\left(1+\frac{\left[M\right]}{M_{L}^{A}}+\frac{\left[M\right]}{M_{R}^{A}}+e^{-\beta \epsilon_{\text{int}}^{A}}\frac{\left[M\right]}{M_{L}^{A}}\frac{\left[M\right]}{M_{R}^{A}}\right)+(1+\frac{\left[L\right]}{L_{I}})\left(1+\frac{\left[M\right]}{M_{L}^{I}}+\frac{\left[M\right]}{M_{R}^{I}}+e^{-\beta \epsilon_{\text{int}}^{I}}\frac{\left[M\right]}{M_{L}^{I}}\frac{\left[M\right]}{M_{R}^{I}}\right)\\ & \approx (1+\frac{\left[L\right]}{L_{A}})\frac{\left[M\right]}{{\tilde{M}}_{L}^{A}}\frac{\left[M\right]}{{\tilde{M}}_{R}^{A}}+(1+\frac{\left[L\right]}{L_{I}})(1+\frac{\left[M\right]}{M_{L}^{I}}+\frac{\left[M\right]}{M_{R}^{I}}+e^{-\beta \epsilon_{\text{int}}^{I}}\frac{\left[M\right]}{M_{L}^{I}}\frac{\left[M\right]}{M_{R}^{I}}). \end{array}$$ (13)

In making these approximations, we have assumed the stricter conditions $e^{2\beta \epsilon }\frac{L_{I}}{L_{A}}⪡1$ and $e^{2\beta \epsilon }\frac{L_{I}}{L_{A}}\frac{M_{X}^{I}}{{\tilde{M}}_{X}^{A}}⪡1$ for the WT, D, and S subunits, all of which are valid assumptions for this system (see S1 Text Section A).

Fig 5 shows the resulting best-fit curves for the anisotropy data, with the corresponding CRP_D/S_ DNA dissociation constants given in Table 2. Since 1 + *r*_0_ ≈ 1, cAMP-unbound CRP binds poorly to DNA, in accordance with the inactive state crystal structure whose DNA recognition helices are buried inside the protein [10]. Additionally, the anisotropy 1 + *r*_1_ = 1.7 of the DNA-CRP-cAMP complex is larger than that of both the cAMP-unbound state and the doubly bound state DNA-CRP-(cAMP)_2_ with 1 + *r*_2_ = 1.4; this suggests that CRP-(cAMP)_2_ binds more weakly to DNA than CRP-cAMP. However, we note that these results depend upon the anisotropy values for the three CRP states (*r*_*j*_ in Table 2); Lanfranco *et al*. assumed that difference between the singly-cAMP-bound CRP state and the unbound CRP state should be the same as the difference between the doubly- and singly-cAMP-bound states and subsequently determined that the singly- and doubly-cAMP bound CRP states bind with roughly the same affinity to DNA. That said, previous studies have supported the claim that the singly-cAMP bound state binds tightest to DNA using multiple experimental methods including proteolytic digestion by subtilisin, chemical modification of Cys-178, and fluorescence measurements [40–42]. Given the ability of the MWC model to characterize the cAMP-binding and DNA-binding data of Lanfranco *et al*., we next consider the final step in the CRP activation cycle, namely, how well CRP can enhance gene expression.

**Table 2:** Parameters for CRP binding to DNA. The anisotropy data for CRP_D/S_ characterized using Eq (9), as shown in Fig 5. Each value is given as a mean ± standard error. The uncertainty in the ${\tilde{M}}_{\text{S}}^{A}$ parameter (shown in Table 1) leads to a corresponding uncertainty in the active CRP dissociation constant *L*_*A*_.

MWC Parameter	Best-Fit Value
*r*_0_, *r*_1_, *r*_2_	{0.1, 0.8, 0.5} ± 0.1
*L*_*A*_, *L*_*I*_	{≤ 30, 30 ± 10} × 10^−9^ M

### Implications of mutations for *in vivo* systems

Since CRP is a global transcriptional activator that governs many metabolic genes in *E. coli* [8], introducing mutations *in vivo* may vastly change cell behavior. Nevertheless, because the framework introduced above is very generic, it can be readily applied to other transcriptional activators that regulate a more limited number of genes. In that spirit, we briefly explore how the CRP mutants characterized in the Lanfranco *et al*. experiments would behave *in vivo* assuming that they only affect a single gene.

#### Simple activation

**Fig 6:**
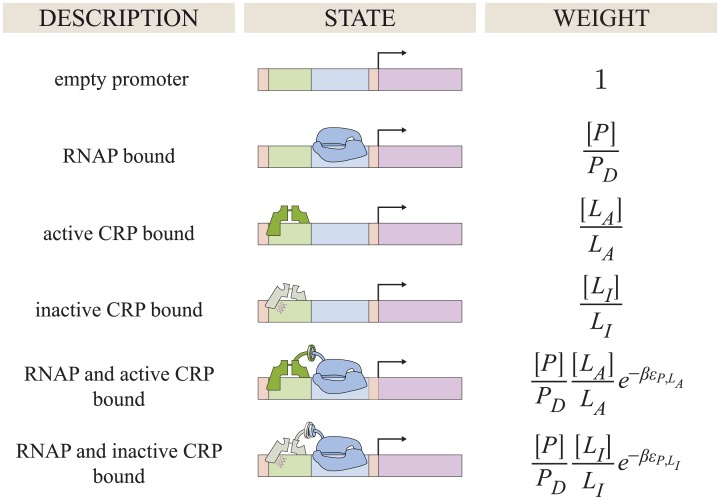
States and weights for a simple activation motif. Binding of RNAP (blue) to a promoter is facilitated by the binding of the activator CRP. Simultaneous binding of RNAP and CRP is facilitated by an interaction energy $\epsilon_{P,L_{A}}$ for active CRP (dark green) and $\epsilon_{P,L_{I}}$ for inactive CRP (light green). cAMP (not drawn) influences the concentration of active and inactive CRP as shown in Fig 4.

Consider a cell with cAMP concentration [*M*] and CRP concentration [*L*] where the population of CRP is split between an active [*L*_*A*_] and an inactive [*L*_*I*_] conformation. Suppose the cell has a concentration [*P*] of RNA polymerase (RNAP) which have a dissociation constant *P*_*D*_ with a promoter of interest. The thermodynamic states of the system are shown in Fig 6, where the activator can bind to and recruit RNAP via an interaction energy $\epsilon_{P,L_{A}}$ between active CRP and RNAP with a weaker interaction $\epsilon_{P,L_{I}}$ between inactive CRP and RNAP. Without these two interaction energies ($\epsilon_{P,L_{A}}=\epsilon_{P,L_{I}}=0$), the RNAP and CRP binding events would be independent and there would be no activation. Moreover, if the two activation energies were the same ($\epsilon_{P,L_{A}}=\epsilon_{P,L_{I}}$), the system could not exhibit the level of activation seen in the data (see S1 Text Section B).

We assume that gene expression is equal to the product of the RNAP transcription rate *r*_trans_ and the probability that RNAP is bound to the promoter of interest, namely,

$$\begin{array}{c} \text{activity}=r_{\text{trans}}\frac{\frac{\left[P\right]}{P_{D}}(1+\frac{\left[L_{I}\right]}{L_{I}}e^{-\beta \epsilon_{P,L_{I}}}+\frac{\left[L_{A}\right]}{L_{A}}e^{-\beta \epsilon_{P,L_{A}}})}{\frac{\left[P\right]}{P_{D}}(1+\frac{\left[L_{I}\right]}{L_{I}}e^{-\beta \epsilon_{P,L_{I}}}+\frac{\left[L_{A}\right]}{L_{A}}e^{-\beta \epsilon_{P,L_{A}}})+1+\frac{\left[L_{I}\right]}{L_{I}}+\frac{\left[L_{A}\right]}{L_{A}}}. \end{array}$$ (14)

**Fig 7:**
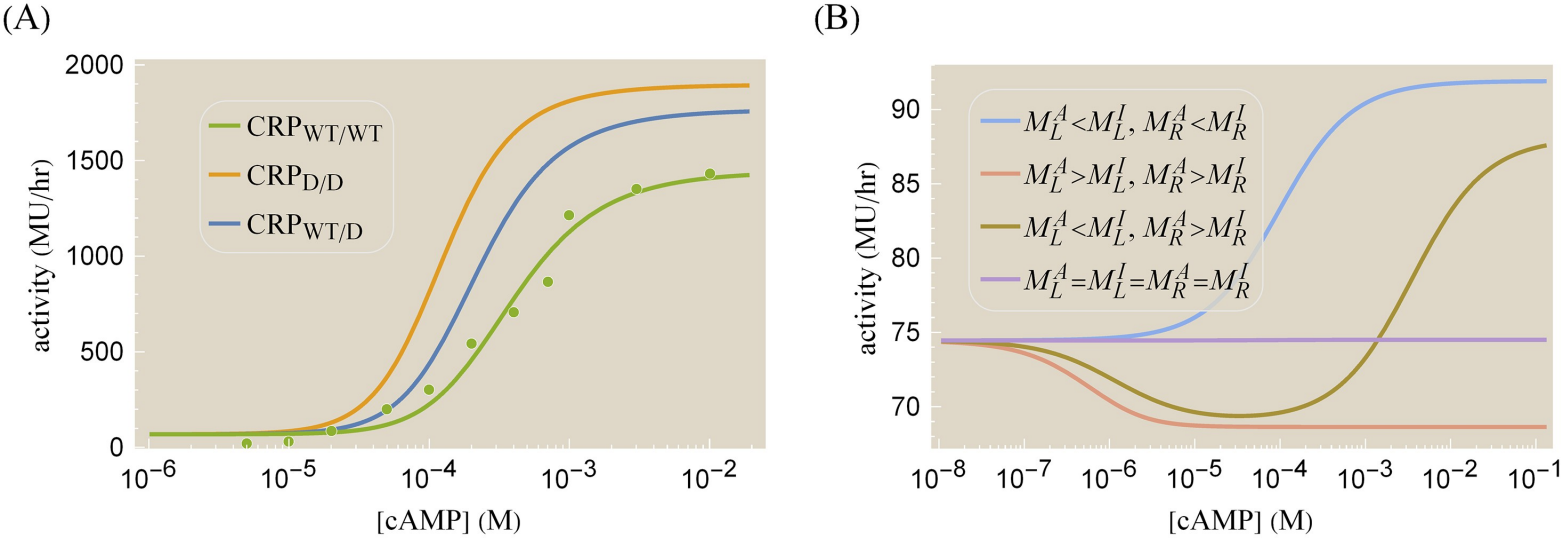
Predicted gene expression profiles for a simple activation architecture. (A) Gene expression for wild type CRP (green dots from Ref. [7]), where 1 Miller Unit (MU) represents a standardized amount of *β*-galactosidase activity. This data was used to determine the relevant parameters in Eq (14) for the promoter in the presence of [*L*] = 1.5 *μ*M of CRP [45]. The predicted behavior of the CRP mutants is shown using their corresponding cAMP dissociation constants. (B) The spectrum of possible gene expression profiles can be categorized based upon the cAMP-CRP binding affinity in each subunit. In all cases, we assumed $M_{L}^{A}=M_{R}^{A}=3\times 10^{-6}\;\text{M}$ and $e^{-\beta \epsilon_{\text{int}}^{A}}=0$. The activation response (blue) was generated using $M_{L}^{I}=M_{R}^{I}=6\times 10^{-6}\;\text{M}$. The repression response (orange) used $M_{L}^{I}=M_{R}^{I}=10^{-7}\;\text{M}$. The peaked response (gold) used $M_{L}^{I}=10^{-7}\;\text{M}$ and $M_{R}^{I}=300\times 10^{-6}\;\text{M}$. The flat response used $M_{L}^{I}=M_{R}^{I}=3\times 10^{-6}\;\text{M}$. The remaining parameters in both plots were $\frac{\left[P\right]}{P_{D}}=130\times 10^{-6}$, $r_{\text{trans}}=5\times 10^{5}\frac{\text{MU}}{\text{hr}}$, *γ* = 0.1, $\epsilon_{P,L_{A}}=-3\;k_{B}T$, $\epsilon_{P,L_{I}}=0\;k_{B}T$, *ϵ* = −3*k*_*B*_*T*, and those shown in Tables 1 and 2.

Several additional factors influence gene expression *in vivo*. First, cAMP is synthesized endogenously by *cyaA* and degraded by *cpdA*, although both of these genes have been knocked out for the data set shown in Fig 7(A) (see Methods and Ref. [7]). Furthermore, cAMP is actively transported out of a cell leading to a smaller concentration of intracellular cAMP. Following Kuhlman *et al*., we will assume that the intracellular cAMP concentration is proportional to the extracellular concentration, namely, *γ*[*M*] (with 0 < *γ* < 1) [43, 44]. Hence, the concentration of active CRP satisfies $\frac{\left[L_{A}\right]}{\left[L\right]}=p_{\text{act}}^{L}\left(\gamma \left[M\right]\right)$ where the fraction of active CRP $p_{\text{act}}^{L}$ is given by Fig 2(A) as

$$\begin{array}{cc} p_{\text{act}}^{L}\left(\left[M\right]\right) & =\frac{1+\frac{\left[M\right]}{M_{L}^{A}}+\frac{\left[M\right]}{M_{R}^{A}}+e^{-\beta \epsilon_{\text{int}}^{A}}\frac{\left[M\right]}{M_{L}^{A}}\frac{\left[M\right]}{M_{R}^{A}}}{1+\frac{\left[M\right]}{M_{L}^{A}}+\frac{\left[M\right]}{M_{R}^{A}}+e^{-\beta \epsilon_{\text{int}}^{A}}\frac{\left[M\right]}{M_{L}^{A}}\frac{\left[M\right]}{M_{R}^{A}}+e^{-2\beta \epsilon }(1+\frac{\left[M\right]}{M_{L}^{I}}+\frac{\left[M\right]}{M_{R}^{I}}+e^{-\beta \epsilon_{\text{int}}^{I}}\frac{\left[M\right]}{M_{L}^{I}}\frac{\left[M\right]}{M_{R}^{I}})}\\ & \approx \frac{e^{-\beta \epsilon_{\text{int}}^{A}}\frac{\left[M\right]}{{\tilde{M}}_{L}^{A}}\frac{\left[M\right]}{{\tilde{M}}_{R}^{A}}}{e^{-\beta \epsilon_{\text{int}}^{A}}\frac{\left[M\right]}{{\tilde{M}}_{L}^{A}}\frac{\left[M\right]}{{\tilde{M}}_{R}^{A}}+(1+\frac{\left[M\right]}{M_{L}^{I}}+\frac{\left[M\right]}{M_{R}^{I}}+e^{-\beta \epsilon_{\text{int}}^{I}}\frac{\left[M\right]}{M_{L}^{I}}\frac{\left[M\right]}{M_{R}^{I}})}. \end{array}$$ (15)

In the last step, we have again introduced the effective dissociation constants from Eqs (3) and (4) and dropped any terms proportional to *e*^*βϵ*^. In addition to these considerations, proteins *in vivo* may experience crowding, additional forms of modification, and competition by other promoters. However, since our primary goal is to understand how CRP mutations will affect gene expression, we proceed with the simplest model and neglect the effects of crowding, modification, and competition.

Because of the uncertainty in the dissociation constant *L*_*A*_ between active CRP and DNA (see Table 2), it is impossible to unambiguously determine the transcription parameters from the single data set for wild type CRP shown in Fig 7(A). Instead, we select one possible set of parameters ($\frac{\left[P\right]}{P_{D}}=130\times 10^{-6}$, $r_{\text{trans}}=5\times 10^{5}\frac{\text{MU}}{\text{hr}}$, *γ* = 0.1, $\epsilon_{P,L_{A}}=-3\;k_{B}T$, and $\epsilon_{P,L_{I}}=0\;k_{B}T$) that is consistent with the wild type data. Next, we inserted the other cAMP-CRP dissociation constants (given in Table 1) into Eq (14) to predict the gene expression profiles of the CRP mutants. Fig 7(A) show the possible behavior of the CRP_D/D_ and CRP_WT/D_ mutants. As expected, replacing a WT subunit with a D subunit shifts the gene expression profile leftwards since the D subunit has a higher cAMP affinity (see Fig 3(A)). Interestingly, the substitution of WT with D subunits comes with a concomitant increase in the maximum gene expression because at saturating cAMP concentrations, a larger fraction of CRP_D/D_ is active compared to CRP_WT/WT_ (96% and 68%, respectively) as seen by using Eq (15) and the parameters in Table 1. Note that we cannot predict the behavior of any of the CRP mutants with S subunits due to the large uncertainty in ${\tilde{M}}_{\text{S}}^{A}$.

Lastly, we probe the full spectrum of phenotypes that could arise from the activity function provided in Eq (14) for any CRP mutant by considering all possible values of the cAMP-CRP dissociation constants $M_{L}^{A}$, $M_{L}^{I}$, $M_{R}^{A}$, and $M_{R}^{I}$ in Eq (15). In particular, we relax our assumption that cAMP binding promotes the CRP’s active state, as a CRP mutation may exist whose inactive state binds more tightly to cAMP than its active state. Fig 7(B) demonstrates that given such a mutation, a variety of novel phenotypes may arise. The standard sigmoidal activation response is achieved when cAMP binding promotes the active state in both CRP subunits ($M_{L}^{A}<M_{L}^{I}$, $M_{R}^{A}<M_{R}^{I}$). A repression phenotype is achieved in the opposite extreme when cAMP binding favors the inactive CRP state ($M_{L}^{A}>M_{L}^{I}$, $M_{R}^{A}>M_{R}^{I}$); we note that the ability to switch between a repressing and activating phenotype was achieved in the Lac repressor with as few as three mutations (see the *R*^*c*^ phenotypes in Ref. [46]). When one subunit is activated and the other is repressed by cAMP ($M_{L}^{A}<M_{L}^{I}$, $M_{R}^{A}>M_{R}^{I}$ or $M_{L}^{A}>M_{L}^{I}$, $M_{R}^{A}<M_{R}^{I}$), a peaked response can form. If the CRP subunits have the same affinity for cAMP in the active and inactive states ($M_{L}^{A}=M_{L}^{I}=M_{R}^{A}=M_{R}^{I}$), then CRP will behave identically for all concentrations of CRP, generating a flat-line response. It will be interesting to see whether these phenotypes can be achieved experimentally.

## Discussion

The recent work of Lanfranco *et al*. provides a window into the different facets of gene regulation through activation [29]. Using insights from their *in vitro* experiments, we can break down the process of activation into its key steps, namely: (1) the binding of cAMP to make the activator CRP competent to bind DNA (Fig 3); (2) the binding of CRP to DNA (Fig 5); and (3) the recruitment of RNAP to promote gene expression (Fig 7(A)). In this work, we generalized the classic MWC and KNF models to include a cAMP interaction energy as well as different DNA-binding affinities for the various cAMP-CRP bound states, allowing us to globally analyze the CRP binding data. Whereas biological research relishes the unique nuances in each system, the physical sciences suggest that common motifs—such as the prevalence of systems adopting an MWC-like description – lead to equally profound insights into the underlying principles governing systems.

By concurrently modeling the multi-step process of activation, we begin to unravel relationships and set strict limits for the binding energies and dissociation constants governing these systems. One hurdle to precisely fixing these values for CRP has been that many different sets of parameters produce the same degenerate responses (see S1 Text Section A). This parameter degeneracy is surprisingly common when modeling biological systems [47, 48], and we discuss how to account for it within the MWC and KNF models of CRP. A key feature of our analysis is that it permits us to identify the relevant parameter combinations for the system, quantify how well we can infer their values, and suggest which future experiments should be pursued to best constrain the behavior of the system.

Lanfranco *et al*. further explored how mutations in one or both subunits of CRP would influence its behavior. Specifically, they used three distinct subunits (WT, D, and S) to create the six CRP mutants shown in Fig 1(B) (black and pink boxes). In this work, we showed that the effects of these mutations can be naturally understood through simple thermodynamic models so that each mutation need not be analyzed individually as if it had no relation to any other mutant. Instead, a compact set of parameters characterizing each subunit (see Table 1) could self-consistently characterize the cAMP-binding of all six mutants. The MWC model was shown to successfully describe the CRP activation data for all mutants whereas the KNF model led to a poorer characterization of the data and moreover incorrectly predicted the inhomogeneous population of CRP in the absence and presence of saturating cAMP. Even though an MWC description of the system was sufficient for the data set considered here, the full CRP system exhibits richer behavior that may require more generalized models that include the ensemble of different states seen by NMR [34, 49]. Nevertheless, it remains a useful exercise to understand how much of a system’s behavior can be successfully captured by such simple models [50].

The models presented here suggest several avenues to further our understanding of CRP. First, we note that both the MWC and KNF models can serve as a springboard for more complex descriptions of CRP or other regulatory architectures [51]. However, a key advantage of simple frameworks lies in their ability to *predict* how different CRP subunits combine. For example, in S1 Text Section B we demonstrate how the data from the three symmetric CRP mutants in Fig 3(A) can be used to coarsely predict the asymmetric mutant responses in Fig 3(B). It would be interesting to see whether such predictions continue to hold as more mutant subunits are characterized, such as for the expanded suite of mutants shown in Fig 1(B). This framework has the potential to harness the combinatorial complexity of oligomeric proteins and presents a possible step towards systematically probing the space of mutations. In addition, any deviations in these predictions will provide further information on how allostery propagates in this system.

Second, several groups have proposed that multiple CRP mutations (K52N, T127, S128, G141K, G141Q, A144T, L148K, H159L from Refs. [9, 32, 52]) only affect the free energy difference *ϵ* between the CRP subunit’s active and inactive states while leaving the cAMP-CRP dissociation constants unchanged. Our model predicts a narrow spectrum of phenotypes for such mutants, since the dependence of the *ϵ* parameter is solely confined to the effective dissociation constants (see Eqs (3) and (4)).

Finally, the framework considered here can be used to predict how the CRP mutants generated by Lanfranco *et al*. would behave *in vivo*. We calibrated the CRP_WT/WT_ gene expression profile using data from Ref. [7] and suggested how the remaining CRP mutants may function within a simple activation regulatory architecture given the currently available data (see Fig 7). It would be interesting to measure such constructs—or better yet, similar activators that regulate very few genes – within the cell and test the intersection of our *in vivo* and *in vitro* understanding both in the realm of the multi-step binding events of transcription factors as well as in quantifying the effects of mutations.

## Methods

As described in Ref. [29], the fractional CRP occupancy data in Fig 3 was measured *in vitro* using 8-anilino-1-naphthalenesulfonic acid (ANS) fluorescence which is triggered by the conformational change of cAMP binding to CRP. Experiments were conducted in 20mM Tris, 50mM NaCl, 1mM EDTA, pH 7.8, and at 25°C. The CRP-DNA anisotropy data in Fig 5 was measured *in vitro* by tagging the end of a 32bp *lac* promoter with a fluorescein molecule and measuring its anisotropy with a spectrometer. When CRP is bound to DNA, anisotropy arises from two sources: the fast bending of the flanking DNA sequence and the slower rotation of the CRP-DNA complex. Sources of error include oligomerization of CRP, the bending of the flanking DNA, and nonspecific binding of CRP to the DNA.

The *in vivo* gene expression data was taken from Kuhlman *et al*. using the *lac* operon *E. coli* strain TK310 [7]. This strain had two genes knocked out: *cyaA* (a gene encoding adenylate cyclase, which endogenously synthesizes cAMP) and *cpdA* (encoding cAMP-phosphodiesterase, which degrades cAMP within the cell). Experiments were done at saturating concentrations of inducer ([IPTG] = 1mM) so that Lac repressor negligibly binds to the operator [53]. In this limit, the only transcription factor affecting gene expression is the activator CRP. Gene expression was measured using *β*-galactosidase activity.

## Supporting information

S1 Text Aforementioned derivations and discussions. (PDF)

S1 File A Mathematica notebook that contains all of the data, reproduces the fitting (using both nonlinear regression and MCMC), and generates the plots in this paper. (PDF)
