# Correction: Theoretical analysis of inducer and operator binding for cyclic-AMP receptor protein mutants

**DOI:** 10.1371/journal.pone.0221295

**Published:** 2019-08-13

**Authors:** The PLOS ONE Staff

S1 File is a duplicate of S1 Text. The publisher apologizes for this error. Please view the correct S1 File below.

## Supporting information

S1 File A Mathematica notebook that contains all of the data, reproduces the fitting (using both nonlinear regression and MCMC), and generates the plots in this paper. (ZIP)
