# Supplementary material for: Theoretical analysis of inducer and operator binding for cyclic-AMP receptor protein mutants
Source: PLoS One. 2018 Sep 26;13(9):e0204275. doi: 10.1371/journal.pone.0204275 (PMC6157895; doi:10.1371/journal.pone.0204275)
Supplement: S1 File — (PDF) [file pone.0204275.s002.pdf]

Supporting Information for A Theoretical Analysis of Inducer and Operator Binding for Cyclic-AMP Receptor Protein Mutants

Tal Einav[1], Julia Duque[2], Rob Phillips[1,3,4*]

1 Department of Physics, California Institute of Technology, Pasadena, CA, United States of America
2 Department of Physics and the London Centre for Nanotechnology, University College London, London, United Kingdom
3 Division of Biology and Biological Engineering, California Institute of Technology, Pasadena, CA, United States of America
4 Department of Applied Physics, California Institute of Technology, Pasadena, CA, United States of America

* phillips@pboc.caltech.edu

A Uncertainties within the Parameter Estimation of CRP

In this section, we examine one major hurdle in fixing the physical parameters of the cAMP-CRP system, namely, that multiple sets of parameters result in identical cAMP binding curves. Fig S1(A) and (B) shows the fractional CRP occupancy Eq (1) made using Parameter Set 1 in Table S1. Similarly, Fig S1(C) and (D) plot the fractional occupancy using Parameter Set 2, yielding an indistinguishable set of curves. We note that both sets of parameters yield curves that are identical to those presented in Fig 3(A) and (B) in the main text, and hence either set could potentially characterize the system.

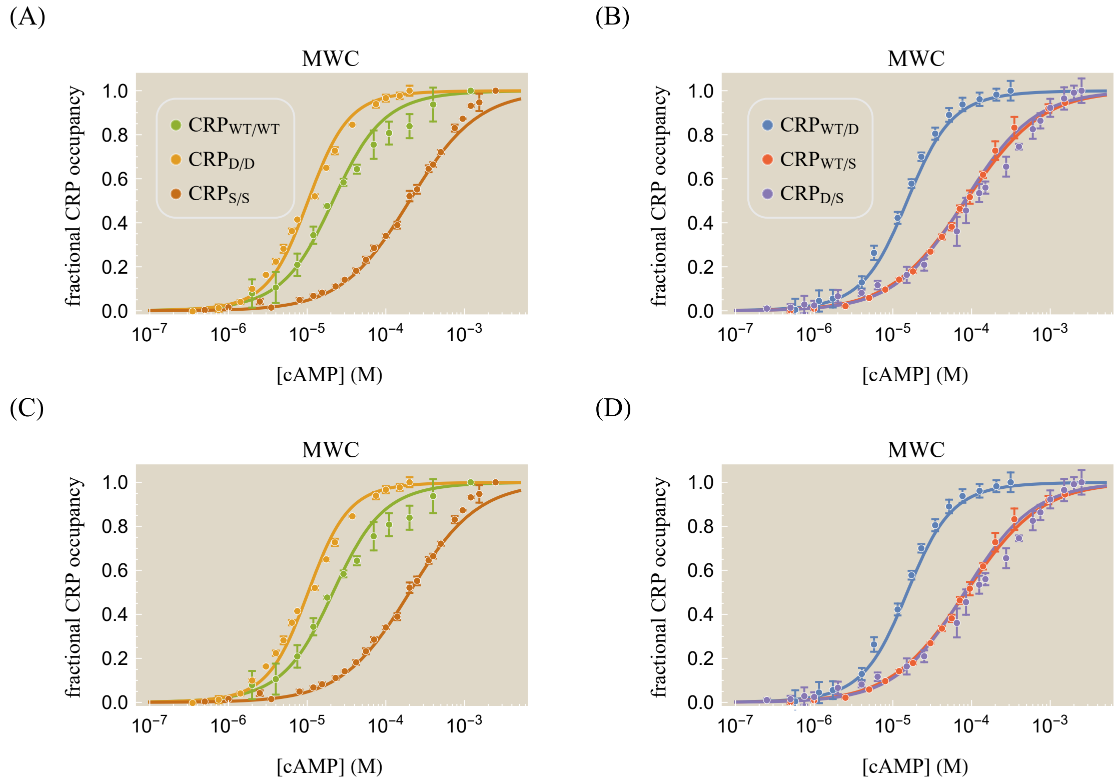

Fig S1. Different sets of parameters yield nearly identical cAMP-CRP binding curves. The same fractional CRP occupancy within the MWC model can be obtained for different parameter values. Panels (A) and (B) were created using Parameter Set 1 from Table S1 while Panels (C) and (D) used Parameter Set 2. Both sets of parameters yield indistinguishable cAMP binding curves.

Given that multiple sets of parameters can fit the data equally well, the precise values of every parameter cannot be uniquely inferred from this set of experiments. Instead, only certain combinations of parameters can be determined. To get rid of this degeneracy, we note that CRP overwhelmingly populates the inactive state in the absence of cAMP, or equivalently that ϵ in Fig 2 is large and negative. Therefore, we consider Eq (1) in the limit $\epsilon \to -\infty$,

Table S1. Parameter degeneracy within the MWC model. The following two sets of parameters yield the same cAMP-CRP binding curves as shown in Fig S1. Note that for each subunit, the product $e^{-\beta\epsilon}e^{-\beta\epsilon_{\text{int}}^A/2}M_X^A$ is the same for both sets of parameter values, in line with Eqs (S2) and (S3).

**Parameter Set 1**		**Parameter Set 2**	
ϵ	$-5\,k_BT$	ϵ	$-10\,k_BT$
ϵ_{int}^A	$0\,k_BT$	ϵ_{int}^A	$0\,k_BT$
ϵ_{int}^I	$0\,k_BT$	ϵ_{int}^I	$0\,k_BT$
M_{WT}^A	170×10^{-9} M	M_{WT}^A	10^{-9} M
M_{D}^A	70×10^{-9} M	M_{D}^A	0.5×10^{-9} M
M_{S}^A	10×10^{-6} M	M_{S}^A	85×10^{-9} M
M_{WT}^I	40×10^{-6} M	M_{WT}^I	40×10^{-6} M
M_{D}^I	50×10^{-6} M	M_{D}^I	50×10^{-6} M
M_{S}^I	200×10^{-6} M	M_{S}^I	200×10^{-6} M

$$\text{fractional CRP occupancy}([M]) = \frac{\frac{1}{2}e^{\beta\epsilon}e^{-\beta\epsilon_{\text{int}}^A/2}\left(\frac{[M]}{\tilde{M}_L^A}+\frac{[M]}{\tilde{M}_R^A}\right)+\frac{[M]}{\tilde{M}_L^A}\frac{[M]}{\tilde{M}_R^A}+\frac{1}{2}\left(\frac{[M]}{M_L^I}+\frac{[M]}{M_R^I}\right)+e^{-\beta\epsilon_{\text{int}}^I}\frac{[M]}{M_L^I}\frac{[M]}{M_R^I}}{e^{2\beta\epsilon}+e^{\beta\epsilon}e^{-\beta\epsilon_{\text{int}}^A/2}\left(\frac{[M]}{\tilde{M}_L^A}+\frac{[M]}{\tilde{M}_R^A}\right)+\frac{[M]}{\tilde{M}_L^A}\frac{[M]}{\tilde{M}_R^A}+\left(1+\frac{[M]}{M_L^I}+\frac{[M]}{M_R^I}+e^{-\beta\epsilon_{\text{int}}^I}\frac{[M]}{M_L^I}\frac{[M]}{M_R^I}\right)}$$
$$\approx \frac{\frac{[M]}{\tilde{M}_L^A}\frac{[M]}{\tilde{M}_R^A}+\frac{1}{2}\left(\frac{[M]}{M_L^I}+\frac{[M]}{M_R^I}\right)+e^{-\beta\epsilon_{\text{int}}^I}\frac{[M]}{M_L^I}\frac{[M]}{M_R^I}}{\frac{[M]}{\tilde{M}_L^A}\frac{[M]}{\tilde{M}_R^A}+\left(1+\frac{[M]}{M_L^I}+\frac{[M]}{M_R^I}+e^{-\beta\epsilon_{\text{int}}^I}\frac{[M]}{M_L^I}\frac{[M]}{M_R^I}\right)}, \quad \text{(S1)}$$

where in the first equality we multiplied the numerator and denominator of Eq (1) by $e^{2\beta\epsilon}$ and defined the effective dissociation constants

$$\tilde{M}_L^A = e^{-\beta\epsilon}e^{\beta\epsilon_{\text{int}}^A/2}M_L^A, \quad \text{(S2)}$$

and

$$\tilde{M}_R^A = e^{-\beta\epsilon}e^{\beta\epsilon_{\text{int}}^A/2}M_R^A, \quad \text{(S3)}$$

while in the latter equality of Eq (2) we neglected all of the terms multiplied by the small quantity $e^{\beta\epsilon}$ (which is equivalent to taking the zeroth order Taylor series about $e^{-\beta\epsilon} \approx 0$). This last assumption is well justified, since both $e^{2\beta\epsilon}$ and $e^{\beta\epsilon}e^{-\beta\epsilon_{\text{int}}^A/2}$ are small, with reported values of the free energy difference between inactive and active CRP ranging from $2\epsilon = -13.7\,k_BT$ [1] to $2\epsilon = -20.7\,k_BT$ [2] while ϵ_{int}^A ranges from $-1\,k_BT$ (slight positive cooperativity) to $4.5\,k_BT$ (strong negative cooperativity) depending on the buffer [3–5]. Importantly, every parameter in Eq (S1) can be determined unambiguously from the data. In other words, for each mutant we can determine the inactive CRP-cAMP dissociation constant for each subunit (M_L^I or M_R^I), the effective active CRP-cAMP dissociation constant ($\tilde{M}_L^A$ or $\tilde{M}_R^A$), and the single value of the inactive cAMP interaction energy (ϵ_{int}^I) which will be the same across all mutants. Indeed, these parameter combinations are the same for both sets of parameters in Table S1.

Lastly, in deriving the approximate form of the anisotropy for the MWC model (Eqs (9)-(13)), we assumed that $e^{2\beta\epsilon}\frac{L_I}{L_A} \ll 1$ and that $e^{2\beta\epsilon}\frac{L_I}{L_A}\frac{M_X^I}{\tilde{M}_X^A} \ll 1$ for the three subunits $X = \{\text{WT}, \text{D}, \text{S}\}$. Since the data cannot fix a precise L_A value for the MWC model (see Table 2), we cannot definitively confirm these inequalities. However, we note that these two relations will hold provided that $\frac{L_I}{L_A} \lesssim 10$ (or $L_A \gtrsim 3 \times 10^{-9}\,\text{M}$), which is a very reasonable bound since nanomolar dissociation constants represent extremely tight binding. In addition, we note that the KNF model has $L_A \approx L_I$ which certainly satisfies the above conditions provided $\epsilon \lesssim -3\,k_BT$.

B Additional Characterizations of the cAMP-CRP Binding Data

In this section, we probe more deeply into the cAMP-CPR data presented in Fig 3 of the main text. We first explore how both the MWC and KNF descriptions for this binding process enable us to use a subset of the available data to predict the remaining data. We then take the opposite approach and analyze each CRP mutant independently within each model and determine how well each data set can conform to an MWC or KNF description. Finally, we touch upon how NMR data allows us to make direct contact between experiment and theory by measuring the fraction of active CRP molecules at saturating cAMP.

B.1 Predicting the Asymmetric Mutants from the Symmetric Mutants

In the main text, our goal was to characterize the full suite of data generated by Lanfranco *et al.* in order to determine the best-possible description of the CRP system. In this section, our goal is not to analyze a system retrospectively, but rather to test how well knowledge of a subset of the CRP mutants can predict the behavior of the remaining mutants. Suppose that Lanfranco *et al.* had only measured cAMP binding for the symmetric CRP mutants (black box in Fig 1(B)). Can we use this data to predict the behavior of the asymmetric mutants (pink box in Fig 1(B))?

Fig S2(A) shows the calibration of the MWC model Eq (1) to the three symmetric CRP mutants which, being the only data fit, are very well characterized. The corresponding parameters are shown in Table S2. Aside from $\tilde{M}^S_{\text{WT}}$ and the interaction energy ϵ^I_{int}, the remaining parameters are all within a factor of 2 of their more precise values obtained by fitting the entire data set (Table 1 in the main text).

Table S2. Best-fit parameters for the symmetric CRP mutant fitting. Using only the symmetric CRP mutant data in Fig S2 allows us to infer the following cAMP-CRP binding parameters from which we can predict the behavior of the asymmetric mutants. These values should be compared with the corresponding best-fit parameters when fitting the entire data set (Table 1).

MWC Parameter	Best-Fit Value	KNF Parameter	Best-Fit Value
$\tilde{M}^A_{\text{WT}}$, M^I_{WT}	$\{20 \pm 2, 20 \pm 3\} \times 10^{-6}$ M	$\bar{M}^A_{\text{WT}}$	$(30 \pm 3) \times 10^{-6}$ M
$\tilde{M}^A_{\text{D}}$, M^I_{D}	$\{10 \pm 1, 20 \pm 3\} \times 10^{-6}$ M	$\bar{M}^A_{\text{D}}$	$(10 \pm 2) \times 10^{-6}$ M
$\tilde{M}^A_{\text{S}}$, M^I_{S}	$\{200 \pm 10, 220 \pm 20\} \times 10^{-6}$ M	$\bar{M}^A_{\text{S}}$	$(250 \pm 20) \times 10^{-6}$ M
ϵ^I_{int}	$2.2 \pm 0.2\, k_B T$	ϵ^A_{int}	$-0.6 \pm 0.2\, k_B T$

Since the three symmetric CRP mutants contain all three types of subunits (WT, D, and S), we can now predict the behavior of the asymmetric CRP mutants without recourse to any further fitting. Using Eq (2) and the parameters in Table S2, the resulting predictions are plotted in Fig S2(B) using dashed lines to emphasize that the data in those plots were not used to fit these curves. Fig S2(C) shows the analogous calibration of the KNF model using the symmetric CRP mutants and Fig S2(D) displays the corresponding predictions of the asymmetric mutants.

As expected, the predicted cAMP-CRP binding curves for both models are worse than the main text fits, since in the main text *all* of the cAMP-CRP data was used to infer the parameters. While these predictions only capture the approximate shape of the data, it must be noted that they provide an answer to the otherwise impossible question: how can you predict the behavior of an asymmetric CRP mutant without measuring it? Note the predictive power scales with the number of subunits. For example, the five symmetric mutants shown in Fig 1(B) can be used to predict the behavior of the ten asymmetric mutants. More generally, given N subunits we could carry out N experiments on the symmetric mutants and predict the responses of the $\frac{N(N-1)}{2}$ asymmetric mutants. Since the number of mutants scales quadratically with N, the number of predictions grows much faster than the number of experiments that must be carried out, enabling us to harness the combinatorial complexity of oligomeric proteins. The expediency of checking the space of mutants theoretically may be well worth the decrease in the quality of this characterization.

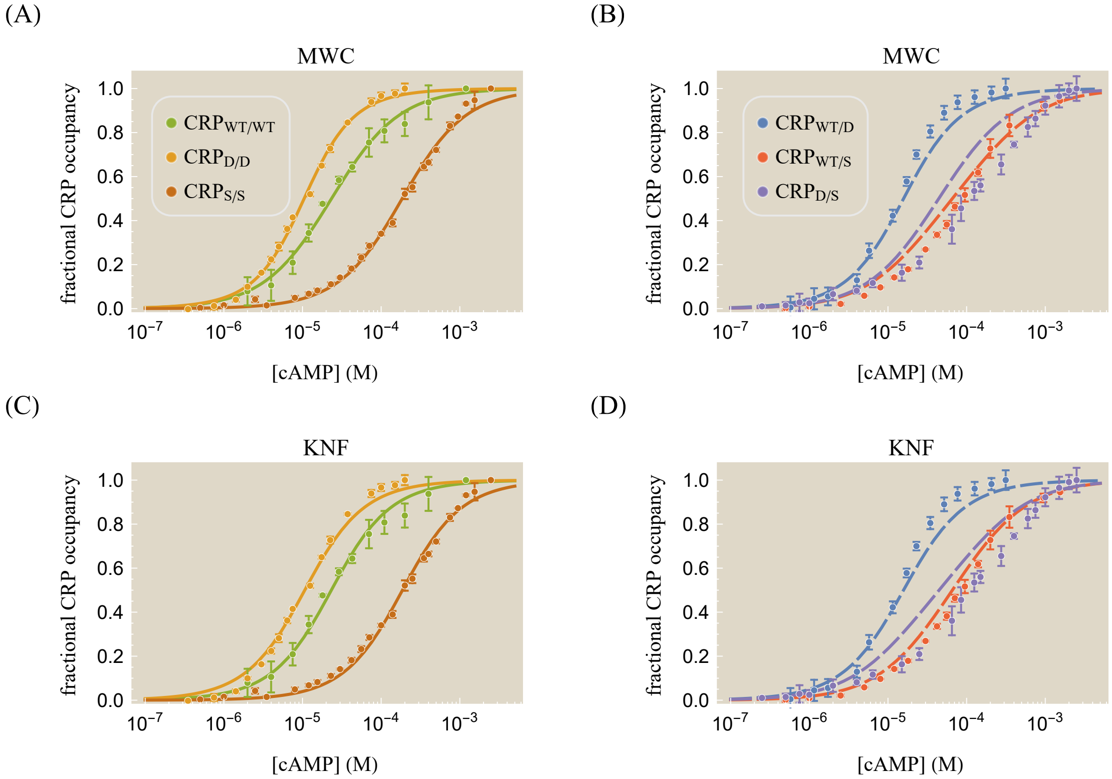

Fig S2. Predicting the behavior of the asymmetric CRP mutants. (A) We infer the cAMP-CRP dissociation constants for the WT, D, and S subunits using the best-fit characterizations (solid curves) of the three symmetric CRP mutants. (B) Using these values, we can predict the behavior of the asymmetric mutants (dashed curves) with no further fitting. (C) In an analogous manner, the KNF model can be calibrated using the symmetric CRP mutants. (D) The corresponding predictions of the KNF model for the asymmetric CRP mutants. The (corrected) sample standard deviations for the asymmetric mutant predictions are 0.08 for both the MWC and KNF models, and the resulting best-fit parameters are shown in Table S2.

B.2 Individual Characterizations of each CRP Mutant

In this section, we relax our assumption that each CRP subunit behaves identically regardless of the composition of its partner subunit and instead analyze a broader question: how well can the MWC or KNF models presented in the text characterize each individual cAMP-CRP binding curve? In other words, suppose there is a complex interaction between CRP subunits, so that the free energy change $\frac{c}{M_{\text{WT}}^{A}}$ of cAMP binding to either subunit in $\text{CRP}_{\text{WT/WT}}$ is different from the corresponding free energy change of the WT subunit in $\text{CRP}_{\text{WT/D}}$. To this end, we fit each CRP mutant's binding data to either the MWC model Eq (1) or the KNF model Eq (5) with no constraint between the parameters of the individual fittings. We note that the original analysis of the six mutants conducted by Lanfranco *et al.* was carried out in an analogous manner by fitting each mutant individually [6].

To simplify this analysis, we restrict ourselves to the case where the two interaction energies are negligible ($\epsilon_{\text{int}}^{A} = \epsilon_{\text{int}}^{I} = 0$); even with this restriction, we find that both the MWC and KNF models can fit the individual data sets remarkably well. In the case where the interaction energies are allowed to be non-zero (not shown), the MWC model negligibly improves while the KNF model becomes indistinguishable from the MWC model. However, we now proceed with the assumption that both interaction energies are vanishingly small.

Fig S3 shows the resulting individual fits for the CRP mutants within the MWC and KNF models,

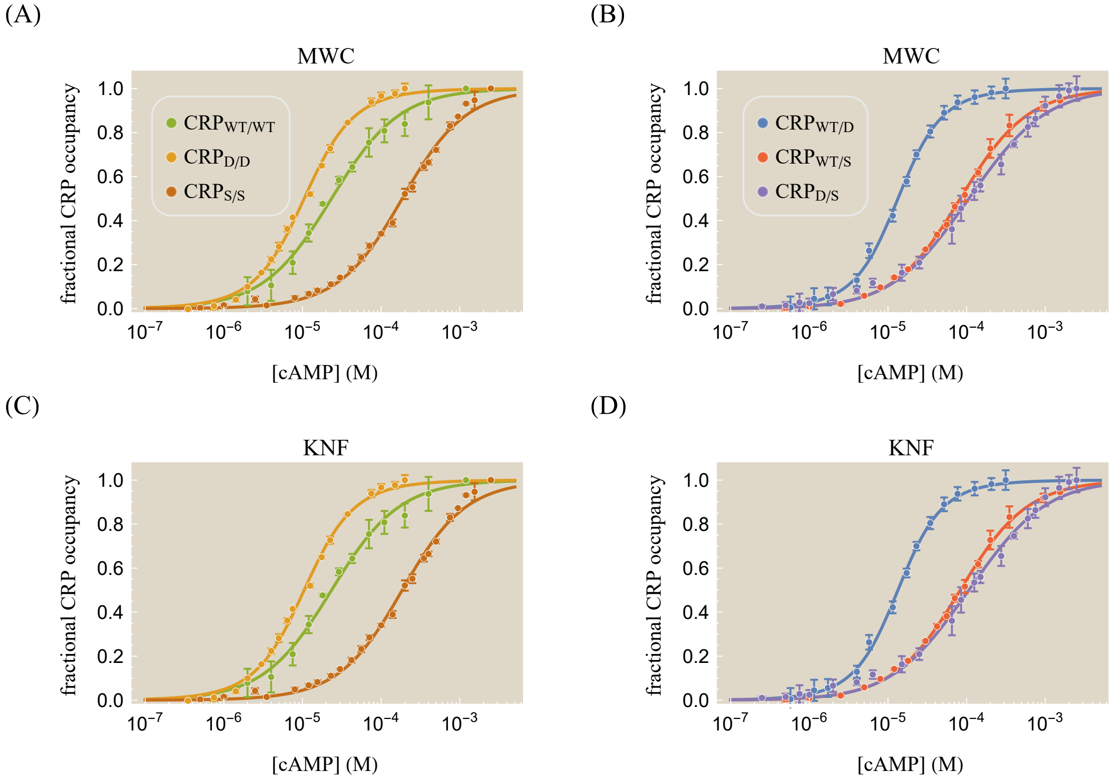

Fig S3. Individual characterization of each CRP mutant. Each of the (A) symmetric and (B) asymmetric CRP mutants are characterized individually using the MWC model Eq (1), showing how closely the model could match the data if the assumption that each subunit behaves identically and independently is relaxed. Similarly, each of the (C) symmetric and (D) asymmetric CRP mutants are characterized separately using the KNF model Eq (5). The sample standard deviation equals 0.02 for the MWC model and 0.04 for the KNF model, and the best-fit parameters for both models are given in Table S3.

with the corresponding parameters given in Table S3. We find that both models can characterize all of the data sets very well; aside from small errors in the KNF description of $\text{CRP}_{\text{WT/D}}$, nearly every data point is within one standard deviation of the predicted value. Thus, both models are capable of characterizing the cAMP-CRP binding behavior, and any discrepancies between the theory and data in Fig 3 may ultimately be attributed to the assumption posited in the text that the WT, D, and S subunits must function identically regardless of the identity of the other CRP subunit. We note that fitting each curve individually results in a sample standard deviation that is a factor of 2 smaller than fitting the six mutants using a single set of parameters (Fig 3); we stress again that this improvement in fit quality comes at the cost of losing both a unified description of the system as well as the predictive power discussed in the previous section.

That said, analyzing each curve individually has the merit in providing the best possible characterization of the data. For example, the individual fitting provides a smooth interpolation between the data points of each CRP mutant, enabling us to compute additional properties of the binding such as the slope at the half-maximal effective concentration (also known as the effective Hill coefficient [7]), which is given by

$$h = \left(2 \frac{d}{d \log[M]} \log \left(\text{fractional CRP occupancy}\right) \right)_{[M]=[EC_{50}^{\text{cAMP}}]}. \tag{S4}$$

Table S4 shows that the effective Hill coefficients of each data set is roughly one. To understand this result, note that the fractional CRP occupancy Eq (6) within the KNF model when both subunits have

Table S3. Best-fit parameters for the individual CRP mutant fitting. The following parameters were determined by fitting each cAMP-CRP data set separately. Thus, each CRP mutant yield slightly different values for the same fit parameters. In both models, the interactions energies are assumed to be zero, $\epsilon^A_{\text{int}} = \epsilon^I_{\text{int}} = 0$.

Mutant Fit	MWC Parameter	Best-Fit Value	KNF Parameter	Best-Fit Value
$\text{CRP}_{\text{WT/WT}}$	$\tilde{M}^A_{\text{WT}}$, M^I_{WT}	$\{210, 20\} \times 10^{-6}$ M	$\bar{M}^A_{\text{WT}}$	20×10^{-6} M
$\text{CRP}_{\text{D/D}}$	$\tilde{M}^A_{\text{D}}$, M^I_{D}	$\{10, 20\} \times 10^{-6}$ M	$\bar{M}^A_{\text{D}}$	10×10^{-6} M
$\text{CRP}_{\text{S/S}}$	$\tilde{M}^A_{\text{S}}$, M^I_{S}	$\{370, 220\} \times 10^{-6}$ M	$\bar{M}^A_{\text{S}}$	180×10^{-6} M
$\text{CRP}_{\text{WT/D}}$	$\tilde{M}^A_{\text{WT}}$, M^I_{WT}, $\tilde{M}^A_{\text{D}}$, M^I_{D}	$\{20, 40, 10, 40\} \times 10^{-6}$ M	$\bar{M}^A_{\text{WT}}$, $\bar{M}^A_{\text{D}}$	$\{10, 10\} \times 10^{-6}$ M
$\text{CRP}_{\text{WT/S}}$	$\tilde{M}^A_{\text{WT}}$, M^I_{WT}, $\tilde{M}^A_{\text{S}}$, M^I_{S}	$\{50, 40, 160, 2000\} \times 10^{-6}$ M	$\bar{M}^A_{\text{WT}}$, $\bar{M}^A_{\text{S}}$	$\{80, 80\} \times 10^{-6}$ M
$\text{CRP}_{\text{D/S}}$	$\tilde{M}^A_{\text{D}}$, M^I_{D}, $\tilde{M}^A_{\text{S}}$, M^I_{S}	$\{90, 40, 160, 1200\} \times 10^{-6}$ M	$\bar{M}^A_{\text{D}}$, $\bar{M}^A_{\text{S}}$	$\{50, 220\} \times 10^{-6}$ M

Table S4. Effective Hill coefficients for the CRP mutants. The effective Hill coefficient Eq (S4) is approximately one for all of the CRP mutants in both the MWC and KNF models.

Mutant Fit	MWC Effective Hill Coefficient	KNF Effective Hill Coefficient
$\text{CRP}_{\text{WT/WT}}$	1.0	1.0
$\text{CRP}_{\text{D/D}}$	1.4	1.0
$\text{CRP}_{\text{S/S}}$	1.1	1.0
$\text{CRP}_{\text{WT/D}}$	1.5	1.0
$\text{CRP}_{\text{WT/S}}$	1.0	1.0
$\text{CRP}_{\text{D/S}}$	0.9	0.9

the same cAMP affinity ($\bar{M}^A_L = \bar{M}^A_R$) is given by the Hill equation

$$\text{fractional CRP occupancy}([M]) = \frac{\frac{[M]}{\bar{M}^A_L}}{1 + \frac{[M]}{\bar{M}^A_L}} \tag{S5}$$

which has an effective Hill coefficient of 1. In other words, if we linearly approximate the fractional occupancy of any curve in Fig S3(C) at its midpoint on the log-linear plot, any symmetric CRP mutant will transition from being unbound to cAMP (fractional CRP occupancy ≈ 0) to mostly bound (fractional CRP occupancy ≈ 1) over approximately one order of magnitude in cAMP concentration.

From Table S3, the three asymmetric CRP mutants also have nearly identical cAMP binding affinities in their two subunits within the KNF model, thereby leading to a value of approximately one for each of their effective Hill coefficients as well. Note that the slopes at the midpoints of the $\text{CRP}_{\text{D/D}}$ and $\text{CRP}_{\text{WT/D}}$ binding curves are slightly steeper than predicted by the KNF model. On the other hand, the effective Hill coefficient for the MWC model, which can be derived in an analogous manner, is more complex than the KNF expression but captures the slopes of all six CRP mutants more accurately, as is seen in Fig S3(A) and (B).

We end with the cautionary note that a Hill coefficient of order unity does not imply that there is little-to-no cooperativity in the system. Indeed, Lanfranco *et al.* determined that $\text{CRP}_{\text{WT/WT}}$ is 15x more cooperative than $\text{CRP}_{\text{S/S}}$ and 5x less cooperative than $\text{CRP}_{\text{D/D}}$, features that are completely masked by only considering the effective Hill coefficient (see the cooperativity (c) column in Table 1 as well as Eq (1) of Ref. [6]). Although the precise definition of cooperativity depends upon the model used, the slope at the half-way point of a sigmoidal response may not be a good indicator of the energies and dissociation constants governing a system.

B.3 Comparing the Fraction of CRP in the Active State

In this section, we derive the fraction of active CRP, enabling us to use NMR measurements to compare the predictions of the MWC and KNF models to experiment. Using Fig 2, the probability that CRP will be in the active state is given by the sum of active weights divided by the sum of all weights, namely,

$$\text{fraction active CRP}([M]) = \frac{1+\frac{[M]}{M_L^A}+\frac{[M]}{M_R^A}+e^{-\beta\epsilon_{\text{int}}^A}\frac{[M]}{M_L^A}\frac{[M]}{M_R^A}}{1+\frac{[M]}{M_L^A}+\frac{[M]}{M_R^A}+e^{-\beta\epsilon_{\text{int}}^A}\frac{[M]}{M_L^A}\frac{[M]}{M_R^A}+e^{-2\beta\epsilon}\left(1+\frac{[M]}{M_L^I}+\frac{[M]}{M_R^I}+e^{-\beta\epsilon_{\text{int}}^I}\frac{[M]}{M_L^I}\frac{[M]}{M_R^I}\right)} \tag{S6}$$

$$\approx \frac{\frac{[M]}{\tilde{M}_L^A}\frac{[M]}{\tilde{M}_R^A}}{\frac{[M]}{\tilde{M}_L^A}\frac{[M]}{\tilde{M}_R^A}+\left(1+\frac{[M]}{M_L^I}+\frac{[M]}{M_R^I}+e^{-\beta\epsilon_{\text{int}}^I}\frac{[M]}{M_L^I}\frac{[M]}{M_R^I}\right)}, \tag{S7}$$

where we have applied the same approximations as in Eqs (2)-(4). Similarly, the fraction of active CRP in the KNF model is given by

$$\text{fraction active CRP}([M]) = \frac{e^{-\beta\epsilon_{\text{int}}^A}\frac{[M]}{M_L^A}\frac{[M]}{M_R^A}}{e^{-2\beta\epsilon}+e^{-\beta\epsilon}\left(\frac{[M]}{M_L^A}+\frac{[M]}{M_R^A}\right)+e^{-\beta\epsilon_{\text{int}}^A}\frac{[M]}{M_L^A}\frac{[M]}{M_R^A}} \tag{S8}$$

$$= \frac{e^{-\beta\epsilon_{\text{int}}^A}\frac{[M]}{\tilde{M}_L^A}\frac{[M]}{\tilde{M}_R^A}}{1+\frac{[M]}{\tilde{M}_L^A}+\frac{[M]}{\tilde{M}_R^A}+e^{-\beta\epsilon_{\text{int}}^A}\frac{[M]}{\tilde{M}_L^A}\frac{[M]}{\tilde{M}_R^A}}, \tag{S9}$$

where we have used Eqs (7) and (8).

Fig 3(C),(F) show the resulting predictions for the fraction of active CRP in both models, with the two models starkly disagreeing in the limit of saturating cAMP. Whereas the KNF model predicts that CRP must ultimately be doubly bound and hence active for sufficiently large concentrations of cAMP, the MWC model purports that some CRP may be inactive regardless of how high the cAMP concentration is raised. In particular, the MWC model predicts that the S subunit is highly biased towards the inactive state, so that only a minute fraction of the S/S mutant will be active at saturating cAMP. This MWC viewpoint agrees with NMR data which shows that only 2% of the S/S mutant is active at saturating cAMP [8].

B.4 CRP Activation with the Same RNAP Affinity in the Active and Inactive CRP States

In this section, we demonstrate why the activation energies between RNAP and active-CRP (ϵ_{P,L_A}) as well as between RNAP and inactive-CRP (ϵ_{P,L_I}) must be different in order to characterize the activation data in Fig 7(A).

To orient ourselves, the maximum possible cAMP activation (see (Fig 6 and Eq (14)) is achieved if in the absence of cAMP all CRP was inactive ($[L_A]=0$, $[L_I]=[L]$) and in the limit of saturating cAMP all CRP would be active ($[L_A]=[L]$, $[L_I]=0$). In such a case, fold-change in activity (i.e., the activity in the presence of saturating cAMP divided by the activity in the absence of cAMP) that we would like to maximize would be given by

$$\text{fold-change in activity} = \frac{\left(1+e^{-\beta\epsilon_{P,L_A}}\frac{[L]}{L_A}\right)\left(1+\frac{[L]}{L_I}+\left(1+e^{-\beta\epsilon_{P,L_I}}\frac{[L]}{L_I}\right)\frac{[P]^2}{P_D^2}\right)}{\left(1+e^{-\beta\epsilon_{P,L_I}}\frac{[L]}{L_I}\right)\left(1+\frac{[L]}{L_A}+\left(1+e^{-\beta\epsilon_{P,L_A}}\frac{[L]}{L_A}\right)\frac{[P]^2}{P_D^2}\right)}. \tag{S10}$$

In the limit where the interactions energies are the same and either really small ($\epsilon_{P,L_A}=\epsilon_{P,L_I}=0$) or really large ($\epsilon_{P,L_A}=\epsilon_{P,L_I}\to-\infty$), the fold change goes to 1 (the only assumption necessary is that $\frac{[P]}{P_D}\ll 1$, as is true in the data set we consider where the best-fit value is $\frac{[P]}{P_D}\approx 10^{-6}$). A in fold-change

emerges at intermediate values of the activation energy, but such a model is unable to fit the 30-fold increase in activation of the experimental data in Fig 7(A).

In the opposite limit where the activation energies are infinitely far apart from each other ($\epsilon_{P,L_A} \to -\infty$ and $\epsilon_{P,L_I} = 0$), the fold-change in activation reduces to the large value of $\left(\frac{[P]}{P_D}\right)^{-2} \approx 10^{12}$, which is more than capable of matching the 30-fold increase in activation seen in the data. As described in the main text, the much more modest energy difference of $\epsilon_{P,L_A} = -3k_BT$ and $\epsilon_{P,L_I} = 0$ can characterize the data.

C Multiple Mutations within a Subunit

In addition to their symmetric mutants (WT/WT, D/D, S/S) and asymmetric mutants (WT/D, WT/S, D/S), Lanfranco *et al.* constructed one additional WT/D+S mutant that had both the D and S mutation within one subunit. The purpose of this mutant was to study the difference between intersubunit communication (D/S) and intrasubunit communication (WT/D+S) in an allosteric protein. In this section, we consider the simplest possible model for this double mutant, namely, that the change in free energy incurred by both the D and S mutations is additive and independent. While mutations are often epistatic (i.e. either not independent or nonlinear; for example see Ref. [9]), this simple model provides a null hypothesis to test whether there are any interactions between such mutations.

Since the WT subunit has already been characterized (see Table 1), we only need to determine how the D+S subunit behaves. To proceed, we will assume that the effects of the D and S mutations on cAMP-CRP affinity are independent as, for example, has been shown to be the case for mutations in the pore region of the nAChR ion channel [10]. More precisely, if the D mutation increases the cAMP-CRP binding energy by $2\,k_BT$ and the S mutation changes it by $-3\,k_BT$, then we assume the D+S mutant changes the cAMP-CRP binding energy by their sum, $-1\,k_BT$. Because dissociation constants are proportional to the exponential of the cAMP-CRP binding energy, $K_D \propto e^{\beta\epsilon_{\text{bound}}}$, the D+S mutation translates into a multiplicative effect on the dissociation constant. For example, the inactive state dissociation constants in the MWC model will obey

$$M^I_{\text{D+S}} = M^I_{\text{WT}} \frac{M^I_{\text{D}}}{M^I_{\text{WT}}} \frac{M^I_{\text{S}}}{M^I_{\text{WT}}}. \tag{S11}$$

In both the MWC and KNF models, we can obtain an analogous expression for the active state dissociation constants by changing $I \to A$ in the superscripts. Multiplying both sides by $e^{-\beta\epsilon}$ leads to the equivalent statement for the effective dissociation constants,

$$\tilde{M}^A_{\text{D+S}} = \tilde{M}^A_{\text{WT}} \frac{\tilde{M}^A_{\text{D}}}{\tilde{M}^A_{\text{WT}}} \frac{\tilde{M}^A_{\text{S}}}{\tilde{M}^A_{\text{WT}}}. \tag{S12}$$

Using these relations, we can predict the behavior of $\text{CRP}_{\text{WT/D+S}}$ without recourse to fitting. Fig S4(A) shows the predictions for the MWC model (dashed black curve) together with the experimental measurements (black data points). For reference, the data and best-fit curves for the three symmetric mutants (green, gold, brown) are also shown. Fig S4(B) demonstrates that a very similar prediction is achieved by the KNF model. In both cases, the experimental measurements roughly follow the theoretical predictions, suggesting that there may be some epistatic interaction in the system, but that assuming linearity and independence for the D and S mutations provides a reasonable zeroth order approximation to the behavior of the system. While we find it interesting that the KNF prediction better matches the $\text{CRP}_{\text{WT/D+S}}$ data, it does not exonerate the KNF model's shortcomings described in the main text (e.g., predicting that $\text{CRP}_{\text{S/S}}$ will be 100% active in the limit of saturating cAMP even though experimental measurements show it to be 2% active in this limit). It would be interesting to compare the ability of the MWC and KNF models to predict the behavior of other double mutants, as would be possible by introducing additional subunit mutations such as G141Q and L148R [11].

With this characterization of the D+S subunit, we can similarly predict how the other possible CRP mutants (D/D+S, S/D+S, D+S/D+S) would behave, as shown in Fig S5. Since Lanfranco *et al.* did not construct any of these mutants, they provide a unique testbed to probe how well the notion of independent mutations holds up within the context of CRP.

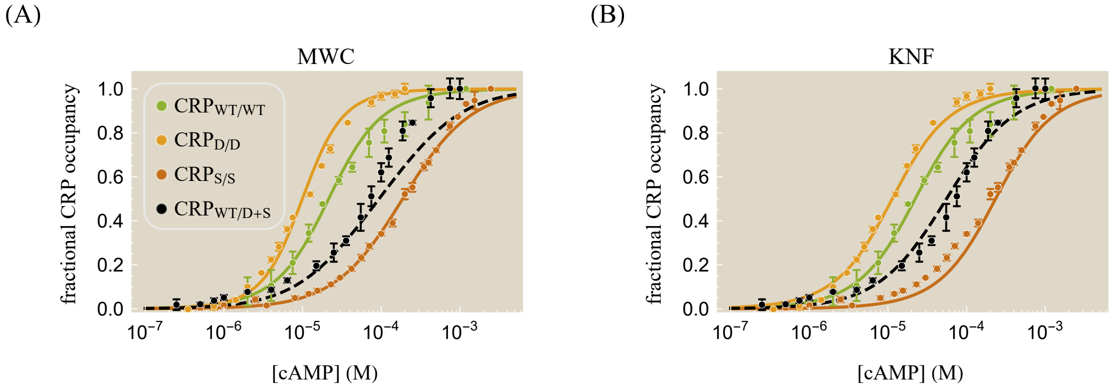

Fig S4. Effect of a D+S double mutation. With both the (A) MWC and (B) KNF models, the D and S mutations are assumed to be independent and additive, leading to the modified dissociation constants given by Eqs (S11) and (S12). The predicted behavior of the D+S subunit (black line, drawn dashed to emphasize that it was not fit to the data) loosely follows the experimental data (black points) for both models. For reference, the symmetric mutants (WT/WT, D/D, S/S) from from Fig 3 are also shown. Parameters used were the same as in Table 1 with no recourse to fitting.

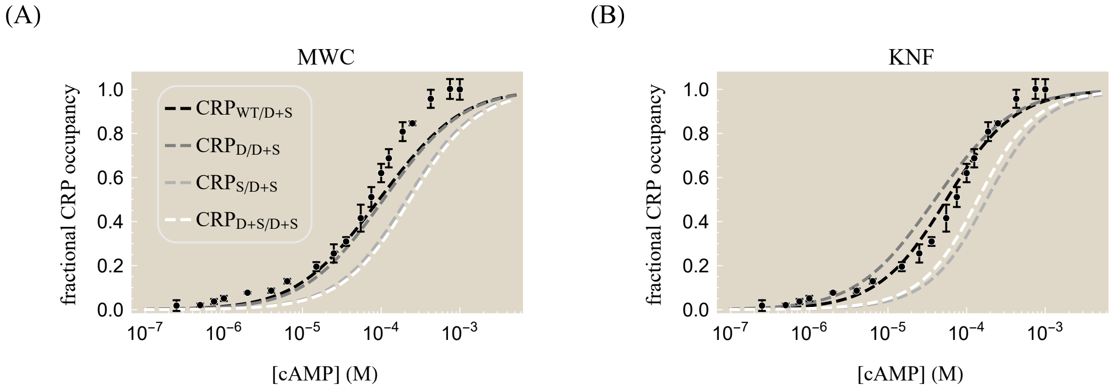

Fig S5. Predicting the behavior of other D+S mutants. Using Eqs (S11) and (S12), the behavior of any CRP mutant with a D+S subunit can be modeled. The four possibilities are shown for the (A) MWC and (B) KNF models together with the data on the WT/D+S mutant. Parameters used were the same as in Table 1 with no recourse to fitting.

References

1. Gunasekara SM, Hicks MN, Park J, Brooks CL, Serate J, Saunders CV, Grover SK, Goto JJ, Lee J-W, Youn H (2015) Directed Evolution of the *Escherichia coli* cAMP Receptor Protein at the cAMP Pocket. Journal of Biological Chemistry 290:26587–26596.
2. Cheng X, Lee JC (1998) Differential Perturbation of Intersubunit and Interdomain Communications by Glycine 141 Mutation in *Escherichia coli* CRP. Biochemistry 37:51–60.
3. Takahashi M, Blazy B, Baudras A, Hillen W (1989) Ligand-modulated Binding of a Gene Regulatory Protein to DNA. Quantitative Analysis of Cyclic-AMP Induced Binding of CRP from *Escherichia coli* to Non-specific and Specific DNA Targets. Journal of Molecular Biology 207:783–796.
4. Popovych N, Sun S, Ebright RH, Kalodimos CG (2006) Dynamically Driven Protein Allostery. Nature Structural and Molecular Biology 13:831–838.
5. Yu S, Maillard RA, Gribenko AV, Lee JC (2012) The N-terminal Capping Propensities of the D-helix Modulate the Allosteric Activation of the *Escherichia coli* cAMP Receptor Protein. The Journal of Biological Chemistry 287:39402–39411.
6. Lanfranco MF, Gárate F, Engdahl AJ, Maillard RA (2017) Asymmetric Configurations in a Reengineered Homodimer Reveal Multiple Subunit Communication Pathways in Protein Allostery. The Journal of Biological Chemistry 292:6086–6093.
7. Marzen S, Garcia HG, Phillips R (2013) Statistical Mechanics of Monod-Wyman-Changeux (MWC) Models. Journal of Molecular Biology 425:1433–60.
8. Tzeng SR, Kalodimos CG (2009) Dynamic Activation of an Allosteric Regulatory Protein. Nature 462:368–372.
9. Rodrigues JV, Bershtein S, Li A, Lozovsky ER, Hartl DL, Shakhnovich EI (2016) Biophysical Principles Predict Fitness Landscapes of Drug Resistance. Proceedings of the National Academy of Sciences 113:E1470–E1478.
10. Auerbach A (2012) Thinking in Cycles: MWC Is a Good Model for Acetylcholine Receptor-channels. The Journal of Physiology 590:93–98.
11. Lin S-H, Kovac L, Chin AJ, Chin CCQ, Lee JC (2002) Ability of *E. coli* Cyclic AMP Receptor Protein To Differentiate Cyclic Nucelotides: Effects of Single Site Mutations. Biochemistry 41:2946–2955.
